# Tracking anharmonic oscillations in the structure of β-1,3-diacetylpyrene

**DOI:** 10.1107/S2052252524010443

**Published:** 2025-01-01

**Authors:** A. Zwolenik, A. Makal

**Affiliations:** a Biological and Chemical Research Centre, Faculty of Chemistry University of Warsaw,Żwirki i Wigury 101 02-089Warszawa Poland; Universidad de Oviedo, Spain

**Keywords:** molecular crystals, dynamical simulations, negative thermal expansion materials, intermolecular interactions, charge spin and momentum densities, quantum crystallography, anharmonic oscillations, 1,3-diacetylpyrene, properties of solids

## Abstract

A recently discovered β polymorph of 1,3-diacetylpyrene has turned out to be a prominent negative thermal expansion material, with a linear thermal expansion coefficient of −199 (6) MK^−1^ at room temperature. Its unique properties can be linked to anharmonic oscillations in the crystal structure that can be attributed to several weak C—H⋯O interactions. The onset and development of anharmonic effects have been successfully tracked over a wide (90–390 K) temperature range using single-crystal X-ray diffraction. Experimental data of sufficient quality combined with Hirshfeld atom refinement of the crystal structures enabled a quantitative analysis of elusive anharmonic effects within the Gram–Charlier formalism. This example highlights that quantum crystallography tools can and should be applied in structural analysis even for data collected at high temperatures as well as of standard (∼0.8 Å) resolution.

## Abstract 2

A recently discovered β polymorph of 1,3-diacetylpyrene has turned out to be a prominent negative thermal expansion material. Its unique properties can be linked to anharmonic oscillations in the crystal structure. The onset and development of anharmonic behavior have been successfully tracked over a wide temperature range by single-crystal X-ray diffraction experiments. Sufficient diffraction data quality combined with modern quantum crystallography tools allowed a thorough analysis of the elusive anharmonic effects for a moderate-scattering purely organic compound.

## 1. Introduction

The term ‘anharmonicity’ refers to the deviation of a system from behaving like a harmonic oscillator. It means that molecular and lattice vibrations cannot be sufficiently described or predicted using a harmonic potential (Attfield, 2018 ▸) and more complicated potentials are required [see Bürgi *et al.* (2000 ▸) and references therein]. Although the ‘an’ prefix suggests some kind of anomaly, anharmonicity of vibrations is quite common; materials (including crystal samples) under experimental conditions will always be ‘anharmonic’ to some extent (Bürgi *et al.*, 2000 ▸; Volkov *et al.*, 2023 ▸). The most prevalent positive anharmonicity comes from thermal expansion, as a result of the diminished strengths of intermolecular interactions and hence reduced frequencies of vibrations. It manifests in the mean-square amplitudes of vibrations larger than expected for a harmonic crystal.

In the case of a less obvious negative anharmonicity, certain intermolecular interactions do not weaken with increased temperature, leading to a decrease in the mean-square amplitudes of certain lattice vibrations. As a consequence, a crystal can shrink in certain directions with heating, displaying negative thermal expansion (NTE), a property much sought after for certain applications (Evans *et al.*, 1997 ▸; Cliffe & Goodwin, 2012 ▸; Attfield, 2018 ▸). It follows that as anharmonicity affects both the amplitude and the frequency of vibrations with respect to harmonic predictions, its presence will influence the stability and mechanical properties of solid materials (Miller *et al.*, 2009 ▸; Attfield, 2018 ▸) as well as carrier mobility in organic semiconductors (Asher *et al.*, 2020 ▸).

As important as the anharmonic effects are, they are inherently difficult to account for in theoretical calculations, requiring a high level of theory and sometimes numerous sequential computations even in quasi-harmonic approximation (Mroz *et al.*, 2021 ▸; Monacelli *et al.*, 2021 ▸). Thus they are hard to model for larger molecular or crystalline systems (*e.g.* most pharmaceuticals) due to computational costs. They should be obtainable with a top-down approach (*i.e.* molecular dynamics), but this is still computationally costly, very dependent on the MD approach taken and thus in need of accurate benchmarking. Hence, experimental techniques such as IR/Raman spectroscopy or diffraction methods, preferably conducted in series at several temperatures, remain the most important source of information on the presence and extent of anharmonic effects. The current work will focus on the X-ray diffraction approach.

### 1.1. Anharmonic vibrations in single-crystal diffraction experiments

Intensities of diffracted radiation are collected over a period of time from a certain volume of crystalline material, providing information on a scattering density averaged over both time and space. This information is interpreted using two components: (*a*) static atomic densities ρ(**r**), calculated with the assumption that no atomic/molecular/lattice vibrations take place; combined with (*b*) atomic probability distribution functions *p*(**u**) describing the mean atomic positions and the deviations from them (**u**). 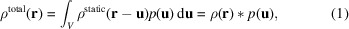
where *p*(**u**) is most often represented as a trivariate Gaussian function that describes the probability distribution for anisotropic harmonic motion in three-dimensional space:
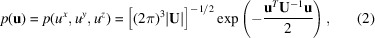
where **U** is the two-dimensional symmetric matrix of mean square atomic displacements 〈*u^i^u^j^*〉 defined in the crystal coordinate axes, 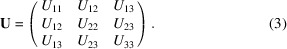
Coefficients *U_ij_* (Å^2^) are anisotropic atomic displacement parameters (ADPs) that are reported in crystallographic files. The **U** matrix can be represented graphically as a probability ellipsoid, centered at the average atomic position, with a surface of constant value *C* such that it encloses the space in which the probability of finding the atom is greater than 50%: 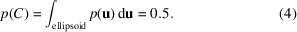
Just as it affects the potential of the oscillator, the anharmonicity of the vibrations changes the shape of the probability distribution of finding an atom around its average position so that it can no longer be described with a simple Gaussian and needs additional terms to describe the deviations from the normal distribution. In the most commonly applied Gram–Charlier (GC) approach (Johnson & Levy, 1974 ▸; Kuhs, 1988 ▸), the anharmonic *p*_anh_(**u**) is approximated in terms of zero and higher derivatives of the normal distribution: 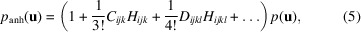
where 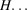 are the Hermite polynomials and functions of displacement coordinates *u^ij^*, whereas the *C_ijk_* and *D_ijkl_* coefficients are related to the moments of *p*_anh_(**u**) and are reported as anharmonic thermal motion coefficients of the third and fourth order accordingly.

The advantage of such a description for the scattering density lies in its generality and flexibility. The new model probability distribution *p*_anh_(**u**) may just be wider than the Gaussian function predicted, or less symmetrical depending on which Hermite functions contribute to it. However, it must be stressed that the *C_ijk_* and *D_ijkl_* coefficients are not easily translated into vibrational frequencies and amplitudes. This would require numerical methods to derive the anharmonic oscillation potential for the purpose of, for example, predicting the exact vibrational spectra at a given temperature. Still, the total *p*_anh_(**u**) can be a useful benchmark against the outcomes of molecular dynamics.

The disadvantage lies in the increased number of parameters to be refined (up to 10 for the third order, 15 for the fourth order), and a risk of over-fitting the model or ‘modeling’ certain effects which are unrelated to vibrations. In particular, the Hermite functions can effectively fit the static scattering density arising from bonding effects, the presence of lone electron pairs, and in particular from occupied *d* and *f* orbitals of the heavier elements (Mallinson *et al.*, 1988 ▸). This ambiguity was identified almost immediately once the tools for modeling bonding density based on diffraction data became available in the form of implementations by Stewart (1976 ▸) and the Hansen–Coppens multipole-model (MM) formalisms (Coppens, 1997 ▸). The MM approach also requires a number of additional parameters to describe static deformation of bonding density. It has been shown that GC coefficients and MM parameters can be strongly correlated (Mallinson *et al.*, 1988 ▸; Meindl *et al.*, 2010 ▸; Herbst-Irmer *et al.*, 2013 ▸). Not accounting for dynamic effects can lead to an unphysical model of the static electron density and *vice versa*.

In the quest for the best experimental models of static bonding density, the focus of the quantum crystallography community was on decoupling the dynamic effects from the static scattering density or minimizing their presence in the experimental data. The latter was usually achievable by conducting experiments at the lowest temperatures possible, thus minimizing the anharmonicity resulting from thermal expansion. The former consisted of collecting diffraction data up to the highest attainable resolution (at least 0.5 Å), thus securing a proper number of experimental data to justify refinement of additional MM or GC parameters, and then refining ADPs, including possibly GC coefficients, against the high-resolution data subset [contributed to more by the cores and hence carrying more information on the atomic vibrations (Hoser *et al.*, 2009 ▸)]. Combining the results of single-crystal X-ray and neutron diffraction should provide the best option, as neutrons are scattered by nuclear density and thus carry information on atomic oscillations unaffected by bonding density. However, data from neutron diffraction studies are very limited due to the availability of facilities and forbidding crystal sizes, and cannot always be successfully combined with X-ray results due to scaling issues.

As a somewhat ironic result, existing reports on ‘modeled’ anharmonic effects in the field of quantum crystallography represent the studies for a subset of materials which could boast good-enough scattering power, often containing heavy elements, investigated at very low temperatures. In fact, such studies avoided the experimental conditions in which anharmonic effects would be of the most importance and systems for which they would be the largest (namely light elements in terminal groups of larger, flexible organic molecules).

The advent of modern quantum crystallography approaches to obtain aspherical atomic scattering factors (AASFs) from aspherical atomic densities, namely the databank approaches ELMAM (Domagała *et al.*, 2012 ▸), UBDB/MATTS (Jha *et al.*, 2022 ▸) or INVARIOM (Dittrich *et al.*, 2013 ▸), followed by Hirshfeld atom refinement (HAR) allowed for effective modeling of bonding densities without over-parametrization of the model (Kulik & Dominiak, 2022 ▸). Over the years, AASF approaches proved their applicability for better experimental geometries of hydrogen atoms (Capelli *et al.*, 2014 ▸; Woińska *et al.*, 2016 ▸; Jha *et al.*, 2020 ▸), especially in specific environments (Woińska *et al.*, 2023 ▸) or structural studies of MOFs (Xu *et al.*, 2023 ▸), or in interpretation of bonding effects in large molecular systems, proteins included (Lecomte *et al.*, 2008 ▸; Malinska & Dauter, 2016 ▸). More importantly, the accessibility of these approaches to users has greatly improved since both databanks and HAR are now executable from within the popular *Olex2* system (Dolomanov *et al.*, 2009 ▸; Kleemiss *et al.*, 2021 ▸; Jha *et al.*, 2023 ▸). With these tools at hand, meaningful refinement of corrections for anharmonic effects in the GC formalism against X-ray diffraction data should be viable even at lower X-ray data resolution (hence, limited number of reflections) and possibly not limited to cryogenic conditions.

The beneficial outcome would be better reliability of the harmonic ADPs obtained in the course of ‘anharmonic’ structure refinement. If unbiased, ADPs can be successfully utilized to derive the thermodynamic properties of materials, allowing us to estimate heat capacities or vibrational contributions to Gibbs free energy and establish the stability order within a polymorphic system (Bürgi *et al.*, 2000 ▸; Hoser & Madsen, 2016 ▸, 2017 ▸).

### 1.2. The example

**Figure 1:**
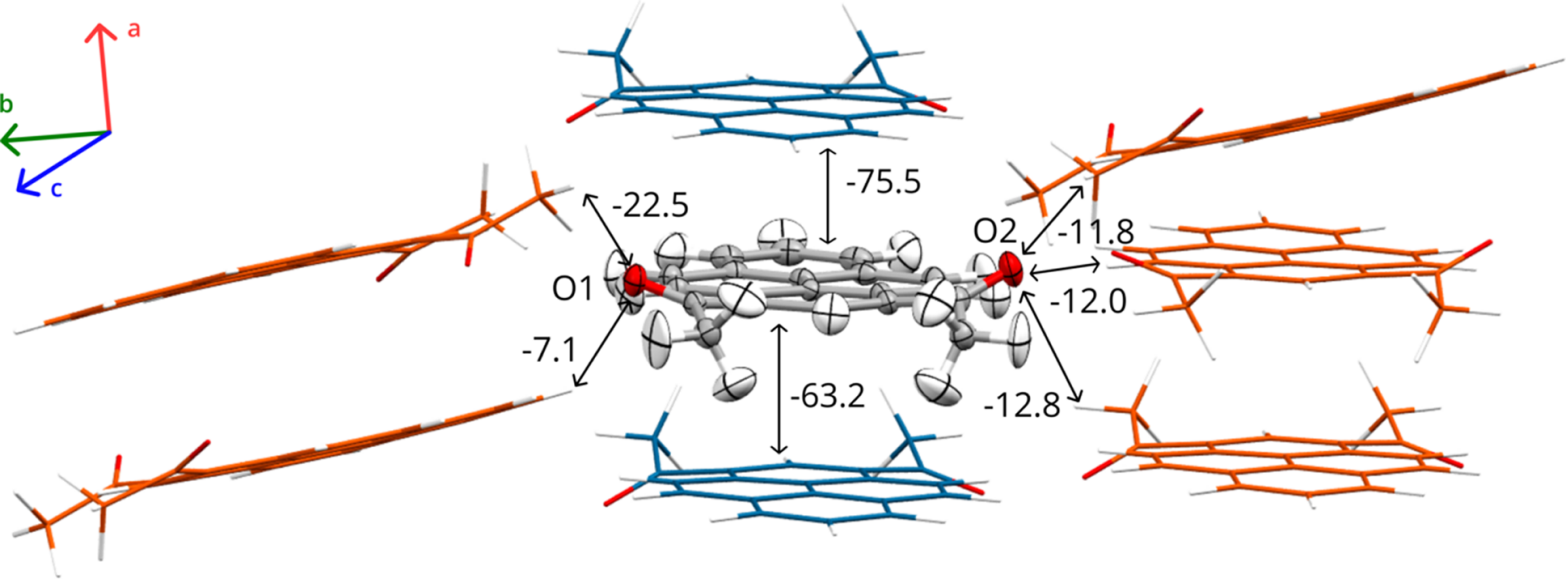
Molecule of 2°AP-β with its closest neighbors in the crystal structure refined at 110 K. Harmonic anisotropic ADPs for the independent molecule are represented at the 50% probability level. Intermolecular interaction energies are given in kJ mol^−1^, as estimated in our former work (Zwolenik *et al.*, 2024 ▸). Symmetry-related molecules involved in π⋯π stacking are shown in blue and those involved in C—H⋯O interactions are shown in orange.

In the present study, we focus on a recently discovered monoclinic polymorph of 1,3-diacetylpyrene, herein denoted 2°AP-β, a luminescent solid-state material built from relatively large (36 atoms) molecular units. The main supramolecular motif in its crystal structure is an infinite π⋯π stacking of inversion-related molecules of 2°AP along [100]. A network of apparently less stabilizing C—H⋯O hydrogen bonds in [101] and [010] is responsible for the inter-stack interactions, as described in our previous work (Zwolenik *et al.*, 2024 ▸) (Fig. 1 ▸).

This crystalline material contains exclusively light elements and does not yield diffraction at resolutions better than 0.6 Å even at cryogenic temperatures, precluding unrestrained experimental charge density refinement. The preparation procedure makes it a non-viable candidate for neutron diffraction studies while the presence of strong and broad orange fluorescence displayed by 2°AP-β made it unsuitable for low-frequency Raman spectroscopy.

2°AP-β has proved challenging when predicting thermodynamic properties based on theoretical periodic DFT calculations (Zwolenik *et al.*, 2024 ▸), indicating that more complicated dynamics take place. 2°AP-β has been shown to display NTE and allowed for anharmonic corrections in the GC formalism to be refined against high-quality X-ray data collected at high pressure (Zwolenik *et al.*, 2024 ▸). All these factors point towards significant anharmonic vibrations occurring around room temperature.

Our goal for the current work was to systematically study the onset and evolution of the anharmonic effects in this material using single-crystal X-ray diffraction conducted under standard laboratory conditions at a wide temperature range up to 390 K. The diffraction experiments would be interpreted by crystal structure refinements including aspherical atomic scattering factors derived from HAR (in order to deconvolute static bonding density features from atomic displacements) and by modeling anharmonic effects with the GC approximation. The statistical significance of the GC coefficients obtained and their meaning in the context of the crystal structure and molecular/lattice vibrations will be discussed.

## 2. Experimental

### 2.1. Sample preparation

1,3-diacetylpyrene was synthesized using the protocol formerly applied for 1,8-diacetylpyrene (Tchoń *et al.*, 2021 ▸). Single crystals of the 2°AP-β polymorph suitable for X-ray diffraction experiments were obtained from a melt according to the procedure described by Zwolenik *et al.* (2024 ▸). The melting point of 2°AP-β was formerly established on a hot stage at 174°C [447 K (Zwolenik *et al.*, 2024 ▸)] and re-established at 177°C with DSC (details provided in Section S10 of the supporting information). DSC did not indicate any phase transitions within the investigated range. For a given variable-temperature X-ray data collection, a single crystal of 2°AP-β was mounted on a nylon loop with a trace of epoxy resin.

### 2.2. X-ray diffraction experiments

The goal was to collect a consistent series of complete, high-quality, standard-resolution X-ray diffraction datasets over a wide temperature range.

Two series of such variable-temperature diffraction experiments were conducted on a Rigaku Oxford Diffraction Supernova using a Cu *K*α X-ray microsource. An Oxford Cryosystem 700 with liquid N_2_ as the coolant was used for temperature control. The initial series covered temperatures from 110 to 230 K: complete redundant datasets have been collected at 10 K intervals. A second series covered temperatures from 190 to 350 K with data collected at 20 K intervals.

Diffraction data collection and reduction were performed with *CrysAlisPro* (Rigaku Oxford Diffraction, 2019 ▸). An anal­ytical absorption correction from the crystal shape was uniformly applied.

Structure determination was performed by intrinsic phasing [*SHELXT* (Sheldrick, 2015 ▸)]. All structures were initially refined using spherical atomic form factors and the least-squares method as implemented in *SHELXL* (Sheldrick, 2008 ▸). All hydrogen atoms could be located directly from the electron density map but their positions and ADPs were refined in the riding approximation (AFIX instructions in *SHELXL*) which restrained C—H distances to prescribed values and the isotropic displacement parameters of hydrogen atoms to those of the *X* atom multiplied by a constant (Sheldrick, 2015 ▸). Note that although ADPs for all atoms in the –CH_3_ groups indicated noticeable rotational oscillation, there were no indications of rotational disorder of the methyl groups, *i.e.* no traces of alternative hydrogen-atom positions in the residual density maps.

In order to obtain a better description of experimental geometry and static electron density distribution, selected structures determined at 110, 150, 190, 250, 290 and 350 K were further refined using aspherical atomic scattering factors derived from HAR using *NoSpherA2*, an implementation of NOn-SPHERical atomic form factors in *Olex2* (Kleemiss *et al.*, 2021 ▸). For each structure, the electron density was calculated from a Gaussian basis set single-determinant SCF wavefunction for a single molecule in an asymmetric unit in the exact experimental geometry. The temperature effects would thus be indirectly manifested in the molecular geometry. Calculations were performed with *ORCA* (version 5.0; Neese *et al.*, 2020 ▸) using the DFT approach with the B3LYP functional, 6-31G(d,p) basis set and Grimme D3 dispersion correction. At this stage, the positions of all atoms including the hydrogen atoms were refined with no restraints. Anisotropic ADPs for hydrogen atoms were also refined for all but the highest temperature (350 K). The ADPs for hydrogen atoms at the highest temperature were refined in isotropic harmonic approximation, with no restraints on their actual values.

For the structures determined at 190, 250, 290 and 350 K, anharmonic corrections in the GC approximation for both oxygen atoms were refined alongside other parameters using *olex2.refine* (Kuhs, 1988 ▸; Volkov *et al.*, 2023 ▸; OlexSys, 2020 ▸).

The quality of the final models and applicability of the anharmonic description of atomic displacements were verified by the fractal plots (Meindl & Henn, 2008 ▸) and estimation of the minimum necessary data resolution (Kuhs, 1988 ▸). Data collection and refinement statistics are summarized in Table 1 ▸, and in Sections S2–S3 of the supporting information. The final structures were deposited with the CCDC Nos. 2379227–2379232 (Table S1.1 of the supporting information). Additionally, the original X-ray diffraction images and associated data are available at https://doi.org/10.18150/SWDNTW from the Repository for Open Data (Interdisciplinary Centre for Mathematical and Computational Modeling, University of Warsaw, Warsaw, Poland; https://repod.icm.edu.pl/). The aspherical atomic scattering factors generated in the course of HARs are also available from RepOD at https://doi.org/10.18150/UDX1VQ.

### 2.3. Periodic DFT calculations

In order to gain insight into the normal vibrational modes of 2°AP-β, periodic *ab initio* DFT calculations were performed using *CRYSTAL17* (Dovesi *et al.*, 2018 ▸) with the B3LYP functional, 6-31 G(d,p) basis set and Grimme D3 dispersion correction (Grimme *et al.*, 2010 ▸, 2011 ▸). The geometry was optimized by adjusting only the coordinates from the experimental geometry with fixed unit-cell parameters, as derived from XRD experiments. The truncation parameters, TOLINTEG, were set to 7 7 7 7 25, and the shrinking factors in the reciprocal space, SHRINK, were set to 8 and 8. Afterwards, normal vibrational modes and their frequencies were calculated at the Γ point of the Brillouin zone using the finite-displacement method. The convergence criteria were set to the default for frequency calculations, using the PREOPTGEOM keyword. Input for *CRYSTAL17* frequency calculations was produced using the *cif2crystal* routine (Madsen, 2006 ▸).

### 2.4. Other approaches

Estimation of linear thermal expansion coefficients based on experimental unit-cell parameters was performed using the *PASCal* server (2024 version; Cliffe & Goodwin, 2012 ▸).

Calculations of approximate intermolecular energies were also performed for high-quality structures resulting from refinements with aspherical atomic scattering factors using *CrystalExplorer* (Mackenzie *et al.*, 2017 ▸) with the DFT method [B3LYP/6-31G(d,p)].

## 3. Results and discussion

### 3.1. Temperature-induced variation in unit-cell parameters

The anomalous thermal expansion of 2°AP-β becomes immediately apparent when inspecting the evolution of unit-cell parameters with increasing temperature. To illustrate this, we combined the results from our previous work (Zwolenik *et al.*, 2024 ▸) with the observations from current complete and redundant experiments.

**Figure 2:**
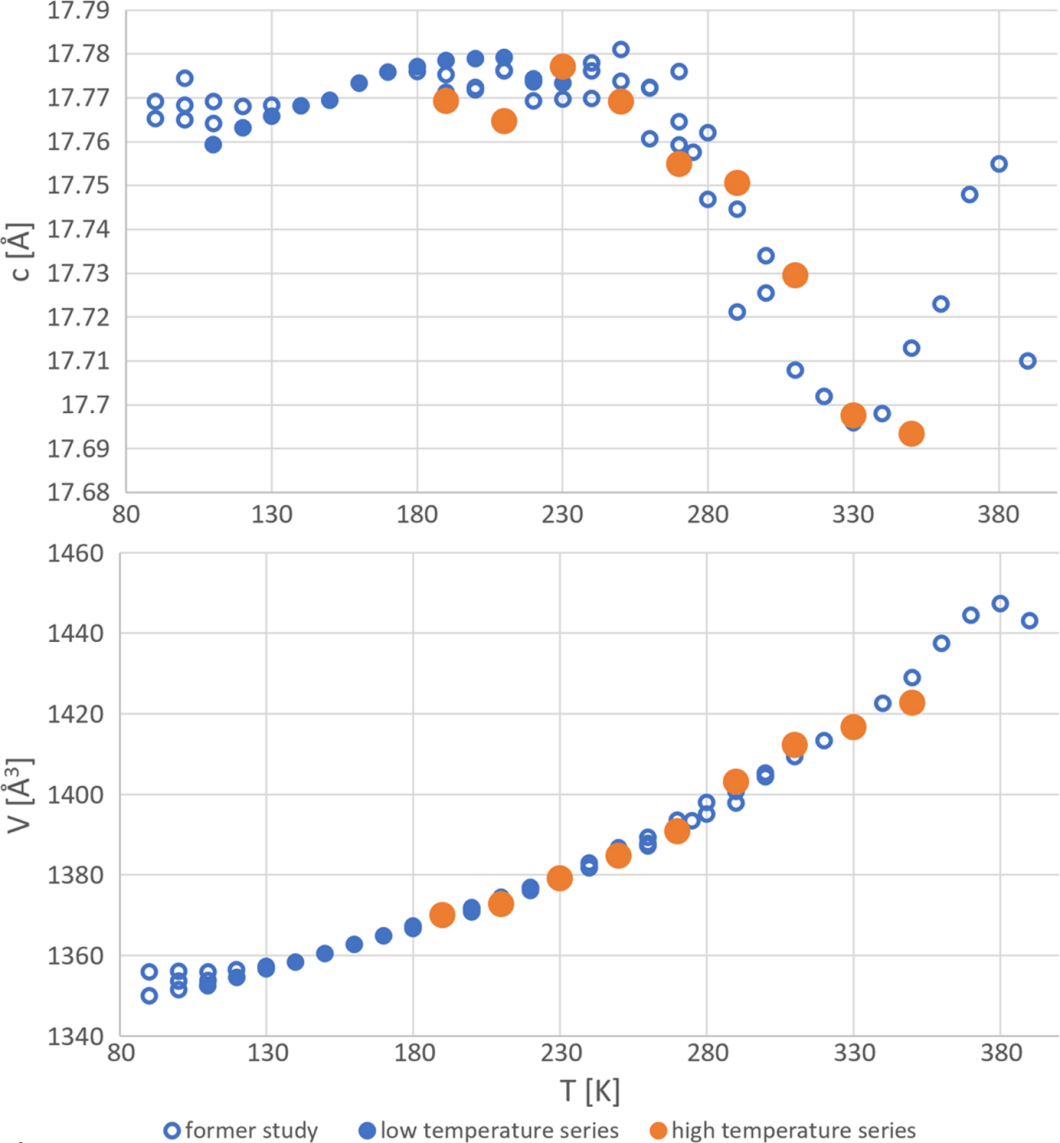
Evolution of the *c* parameter and unit-cell volume with temperature. The empty markers refer to datasets collected during a former study (Zwolenik *et al.*, 2024 ▸), full markers represent data related to the current research. Data for which structures have been solved, refined and discussed in detail in the current study are highlighted in orange.

Although normal thermal expansion should lead to increased lengths of all lattice constants, the *c* parameter retains almost the same value below 210 K and decreases rapidly above it and up to about 350 K (Fig. 2 ▸).

**Figure 3:**
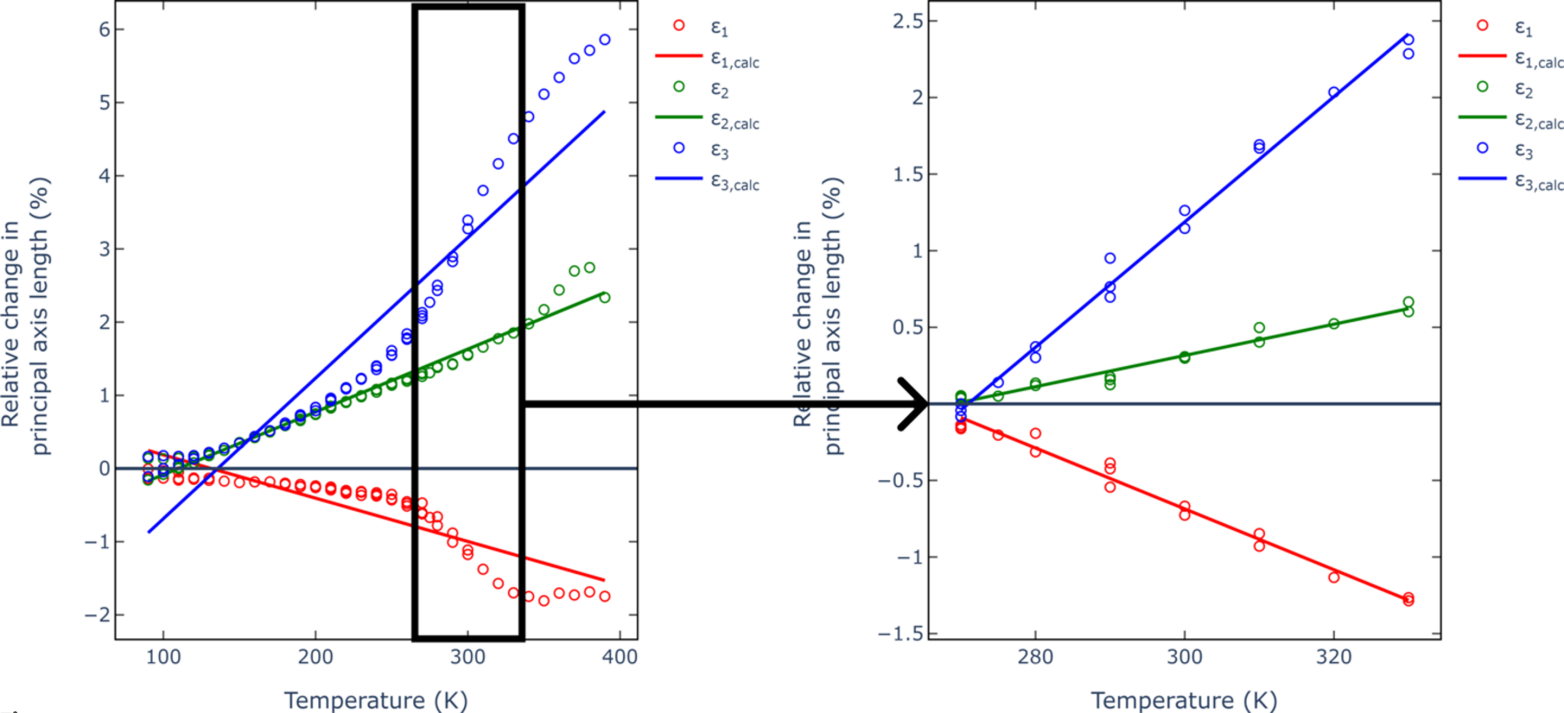
Evolution of Δ*L*/*L* with temperature in 2°AP-β along the principal axes of the thermal expansion tensor, namely along the [010] (blue), [100] (green) and approximately [101] (red) crystallographic directions. Solid lines represent the closest linear fits for the Δ*L*/*L* versus Δ*T* relations. The inset on the right represents the temperature range where the changes are strictly proportional to *T*. Illustrations generated using the *PASCaL* server (Cliffe & Goodwin, 2012 ▸).

This anomalous effect becomes even more apparent once the changes are analyzed along the principal directions of the thermal expansion tensor. In the monoclinic system with the standard choice of the [010] direction as unique, one of the principal directions will align exactly with the unique direction, while the remaining two may or may not align with crystallographic directions. Indeed, in the case of 2°AP-β, the analysis with *PASCaL* indicated the principal directions of the thermal expansion as aligning with [010] (X3), [100] (X2) and approximately [101] (X1). Considered in such a reference frame, the anomalous behavior of the material in the X1 direction is unequivocal (Fig. 3 ▸).

**Figure 4:**
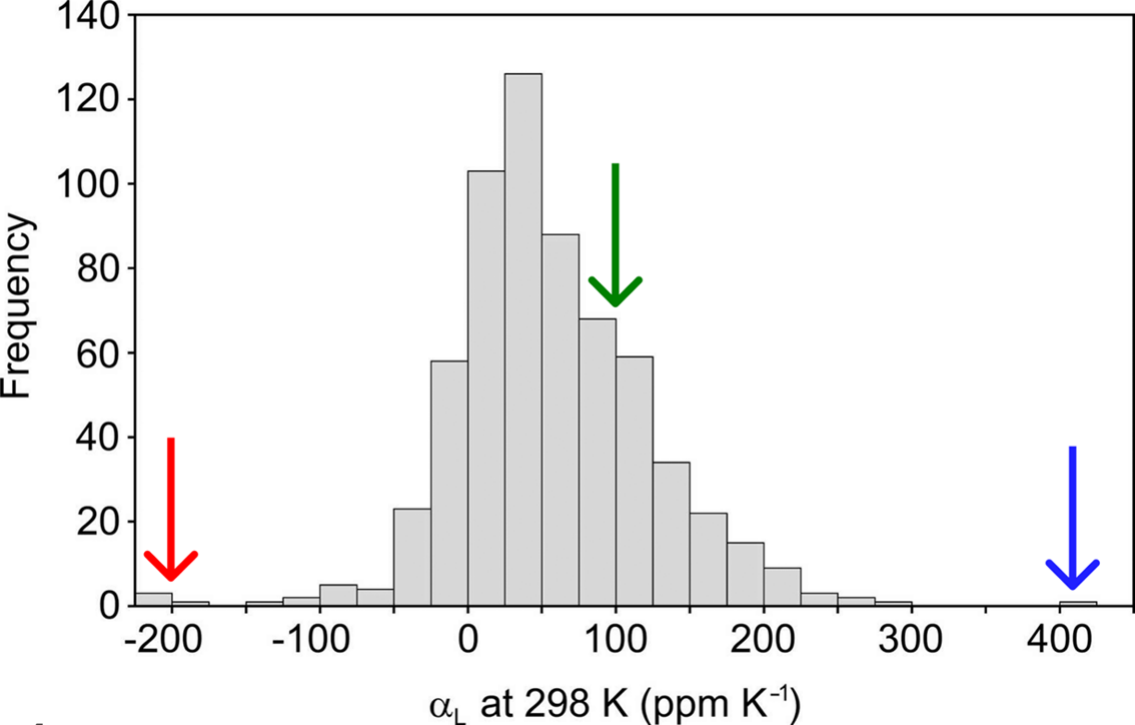
Histogram of the principal expansion coefficients (α_*L*_) at 298 K derived from the 210 reliable structure families, reproduced from the work of Bond (2021 ▸). The α_*L*_ coefficients obtained for 2°AP-β along the [010] (blue), [100] (green) and approximately [302] (red) crystallographic directions are marked on the histogram as arrows.

Thermal expansion is quantified through the linear thermal expansion coefficient, α_*L*_:
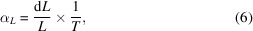
which measures the change in length *L* of an object with temperature *T* along the chosen direction. The formerly reported value of the NTE coefficient α_*L*_ = −56 (6) MK^−1^ (Zwolenik *et al.*, 2024 ▸) was estimated in a temperature span from 90 to 390 K. Closer inspection of the thermal expansion along the principal direction clearly indicates that a single linear fit is inappropriate for the whole temperature range. The anomalous behavior of 2°AP-β appears the most pronounced between 270 and 330 K, where the linear fit to the Δ*L*/*L* versus Δ*T* relation is decidedly more appropriate. The NTE coefficient determined at such *T* range is −199 (6) MK^−1^, while the maximum expansion coefficient is 409 (8) MK^−1^ (Table 2 ▸). According to the survey performed by Bond (2021 ▸) on organic structures deposited in the CSD database (Groom *et al.*, 2016 ▸), such thermal expansion coefficients are exceptional for a purely organic compound (Fig. 4 ▸), and thus highly meaningful.

### 3.2. Effects of anharmonicity in residual density maps

The exceptional anomalous linear thermal expansion of 2°AP-β as well as our former study on its pressure-induced behavior clearly indicated that substantial anharmonic effects may be involved. Their presence should be manifested in residual density maps for the refined crystal structures once the static deformation density of a molecule has been properly modeled by applying AASFs in the crystal structure refinement. We tested this assumption by performing HARs for 2°AP-β against data from a sub-series of temperatures.

**Figure 5:**
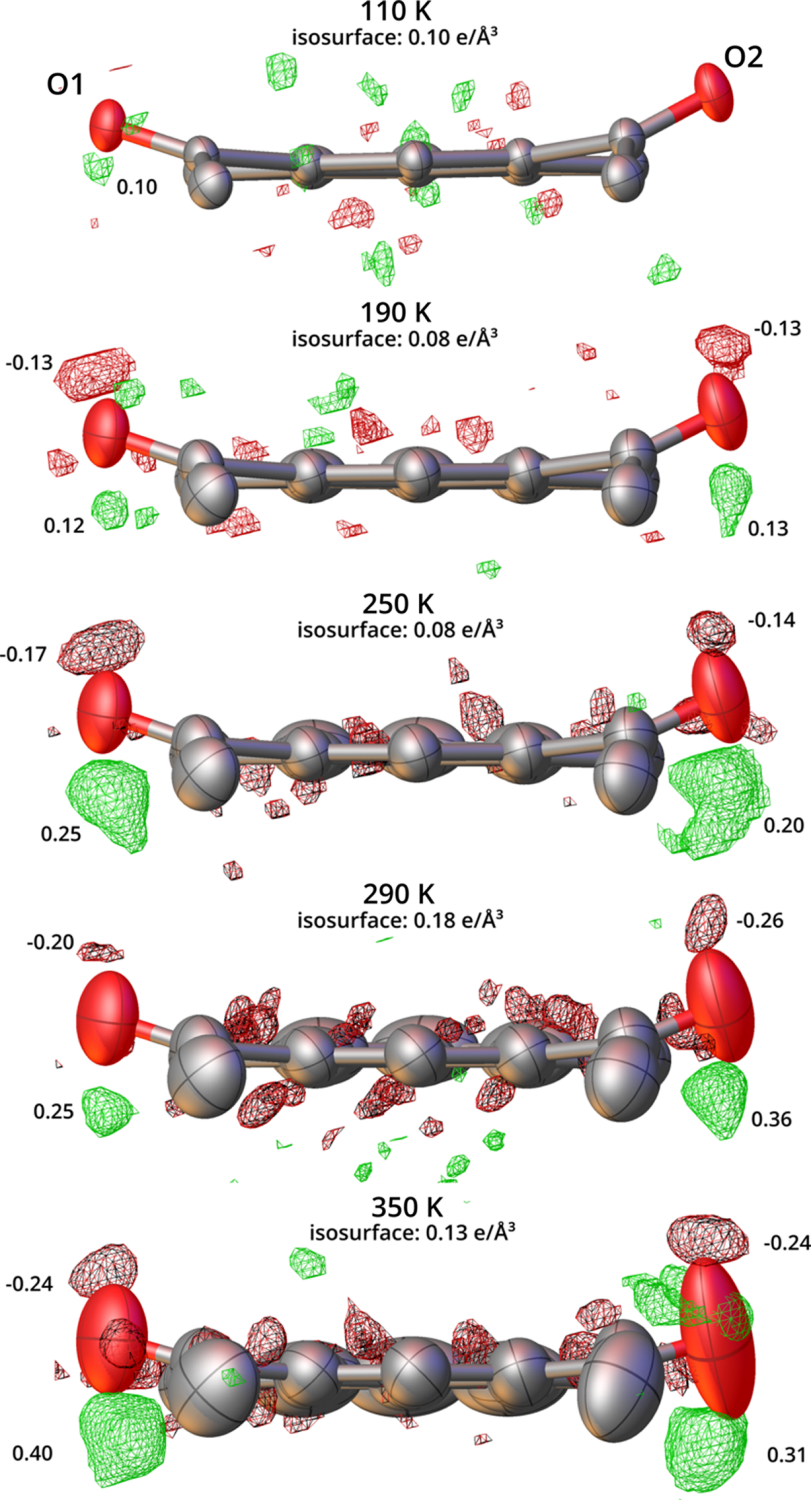
Residual density features in the structures of 2°AP-β after HARs against data at selected temperatures. View along the long axis of the pyrene molecule. ADPs are represented at the 50% probability level, with hydrogen atoms omitted for clarity. Red and green isosurfaces represent the negative and positive values of the residual density at the given isovalue, respectively. Maximum and minimum residual density features at each oxygen atom are indicated accordingly.

After HAR there remained indeed unmodeled residual density features present near both oxygen atoms, indicative of the additional out-of-pyrene-plane oscillations of the carbonyl groups (Fig. 5 ▸). Note that the observed features were small, not exceeding 0.4 e Å^−3^, and could easily be overlooked in the course of the standard crystal structure determination. They would not, for instance, raise serious *checkCIF* alerts (Spek, 2009 ▸, 2020 ▸). Nevertheless, they appeared consistently across several experimental series and for different crystal specimens and their extent increased steadily with increasing temperature which suggested that they were caused by either a dynamic disorder or anharmonic oscillations of carbonyl groups. The presence of anomalous thermal expansion in the material prompted us to pursue the anharmonic oscillation hypothesis.

#### 3.2.1. Test against the Kuhs’ rule

With anharmonic oscillation features being so subtle and prone to being mistaken with other effects, we had to verify whether they could truly be observable and interpretable with our experimental data.

**Figure 6:**
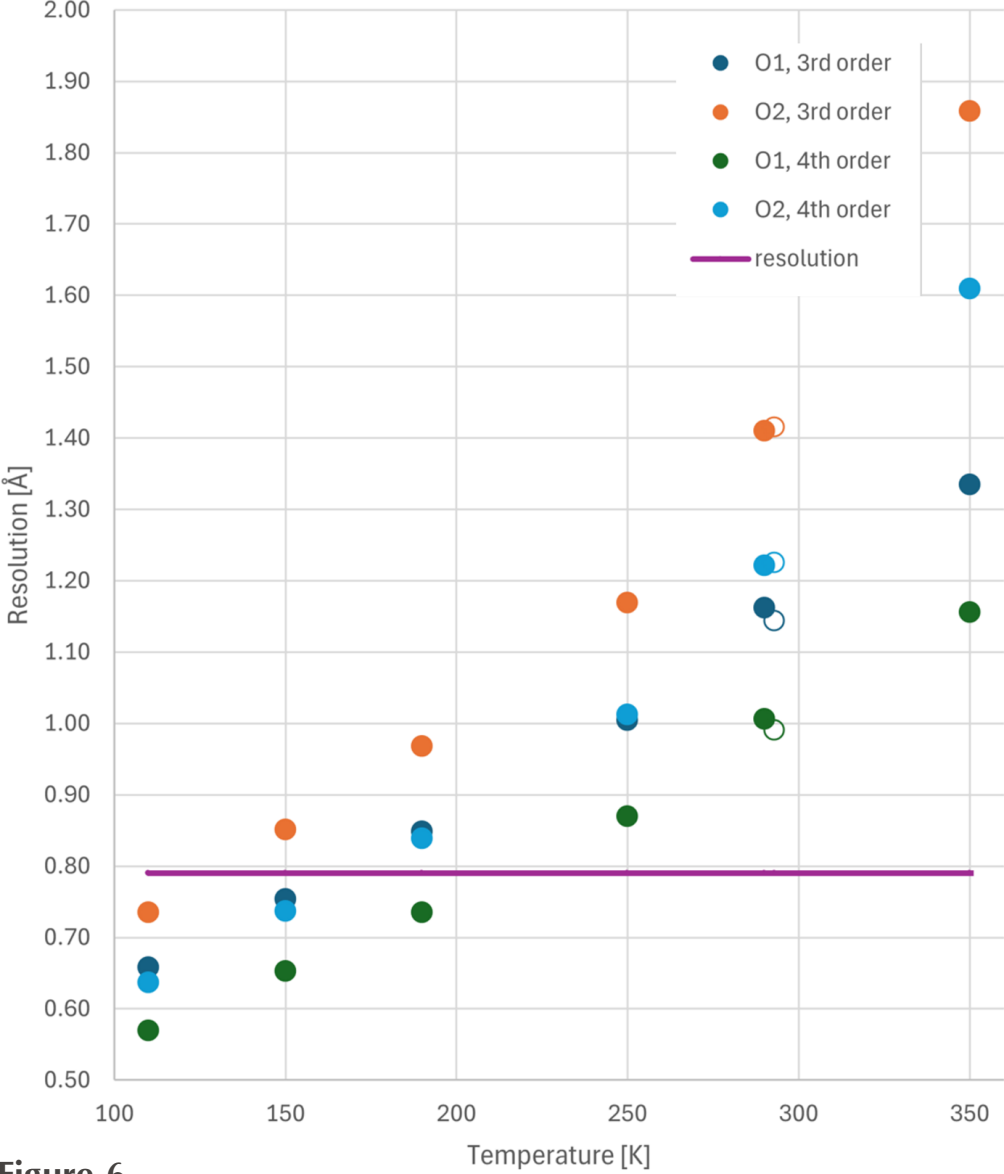
The minimum resolution required, according to Kuhs’ formula, to meaningfully refine corrections for anharmonic oscillations in the GC formalism for O1 and O2 atoms in 2°AP-β as a function of temperature. The experimental resolution limit for the Cu *K*α diffraction data is marked as a purple line. Empty points refer to the data collected at 293 K in our former study (Zwolenik *et al.*, 2024 ▸).

The minimum resolution, *h_n_* (Å^−1^), of diffraction data necessary to introduce corrections for anharmonic oscillations in the GC formalism of an *n*-th order for a given atom can be calculated from a formula proposed by Kuhs (1988 ▸) from harmonic *u*^2^ mean-square displacement parameters of that atom:
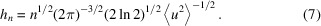
In the case of diffraction data collected in this study, their resolution was limited using the Cu radiation source to 0.78 Å (0.64 Å^−1^). Nevertheless, such resolution turned out to be sufficient to perform anharmonic refinement of third- and fourth-order GC coefficients of both oxygen atoms above 190 K (Fig. 6 ▸, details in Section S4 of the supporting information). Although there were not enough data to perform a reliable refinement of fourth-order GC coefficients for O1 at 190 K, it will be shown below that such parametrization was not necessary.

### 3.3. Modeling anharmonicity

#### 3.3.1. Reliability of the corrections for anharmonicity

**Figure 7:**
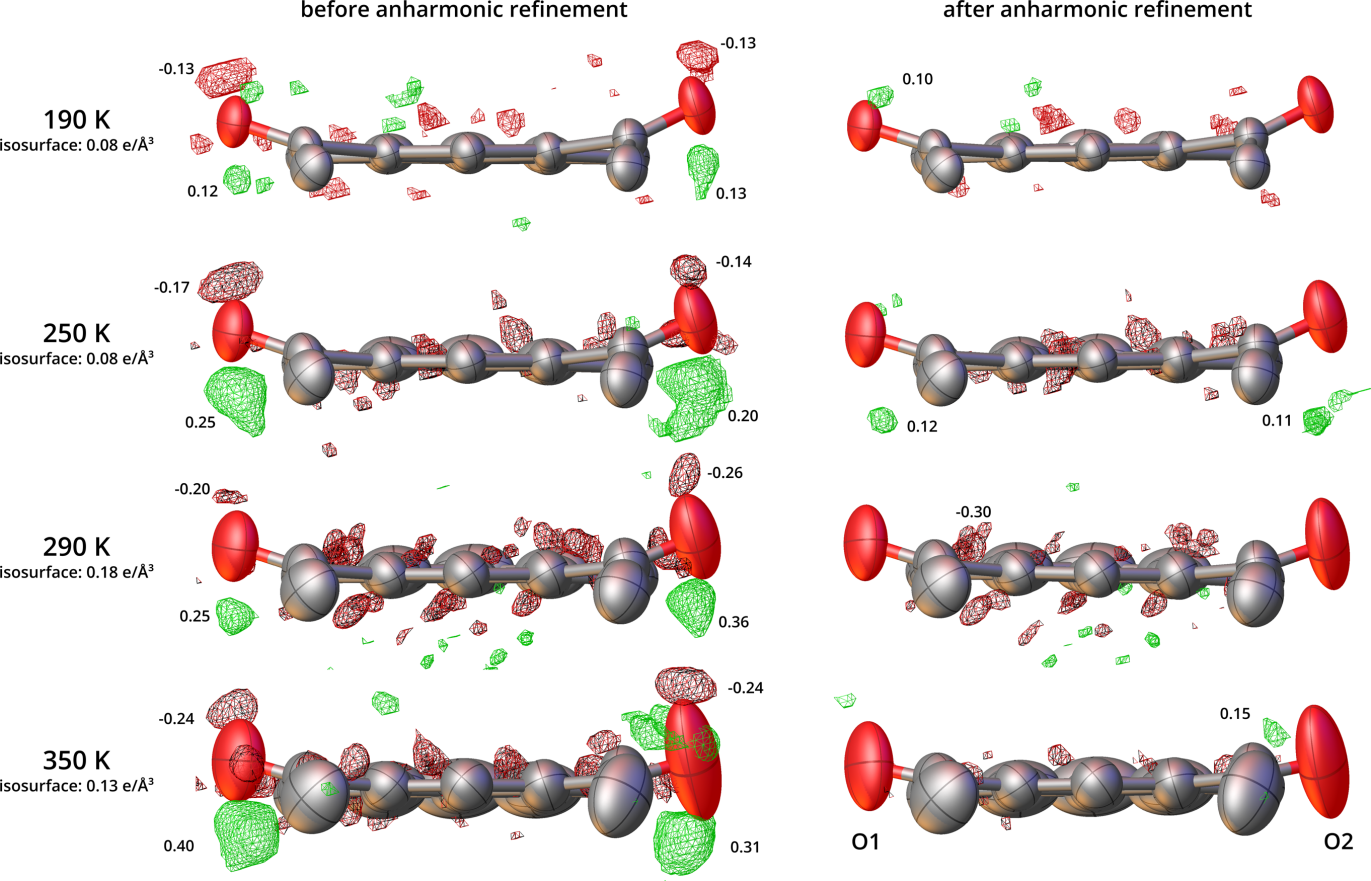
Residual density features in the structures of 2°AP-β after HARs against data at selected temperatures before (left) and after (right) introducing the corrections for the anharmonic oscillations in the GC formalism. View along the long axis of the pyrene molecule. Atomic harmonic displacement parameters are represented at the 50% probability level, with hydrogen atoms omitted for clarity. Red and green isosurfaces represent the negative and positive values of the residual density at the given isovalue, respectively. Maximum and minimum residual density features at each oxygen atom are indicated accordingly.

**Figure 8:**
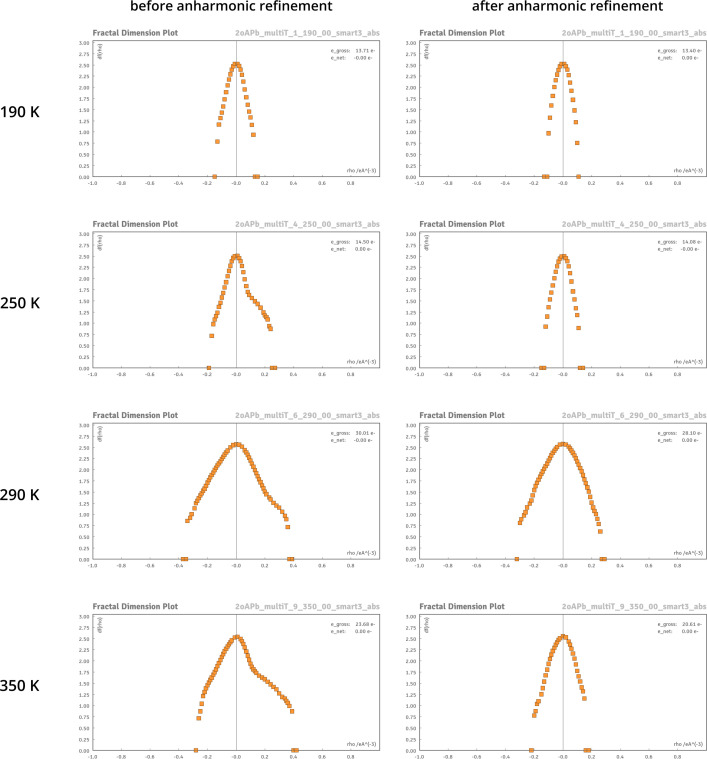
Fractal dimension plots (Meindl & Henn, 2008 ▸) for structures refined without (left) and with (right) anharmonic atomic displacement corrections in the GC formalism. Ideal parabolic shapes suggest a random distribution of residual density (*i.e.* a structural model well fitted to the experimental data).

Structure refinements using the HAR approach with corrections for anharmonic oscillations proved fully justified at all tested temperatures (from 190 to 350 K). In all instances, *R* factors were improved after introducing corrections for anharmonic oscillations (Table 1 ▸). Characteristic residual density features disappeared (Fig. 7 ▸), and the residual density distributions became decidedly more random, as shown in fractal dimension plots (Figs. 8 ▸ and S5.1) even at 350 K.

More importantly still, the GC coefficients refined against data collected at various temperatures appear quite reliable. As expected, only selected GC coefficients refined to statistically significant values, *i.e.* above three estimated uncertainties (detailed in Section S6 of the supporting information). At 190 K (*i.e.* at the lowest temperature at which the residual density features related to anharmonicity could be distinguished), only selected third-order GC coefficients were statistically significant. This was in agreement with the outcome of the Kuhs’ test, which indicated that refinement of the fourth-order GC parameters would be meaningless at that temperature and that particular X-ray data resolution.

**Figure 9:**
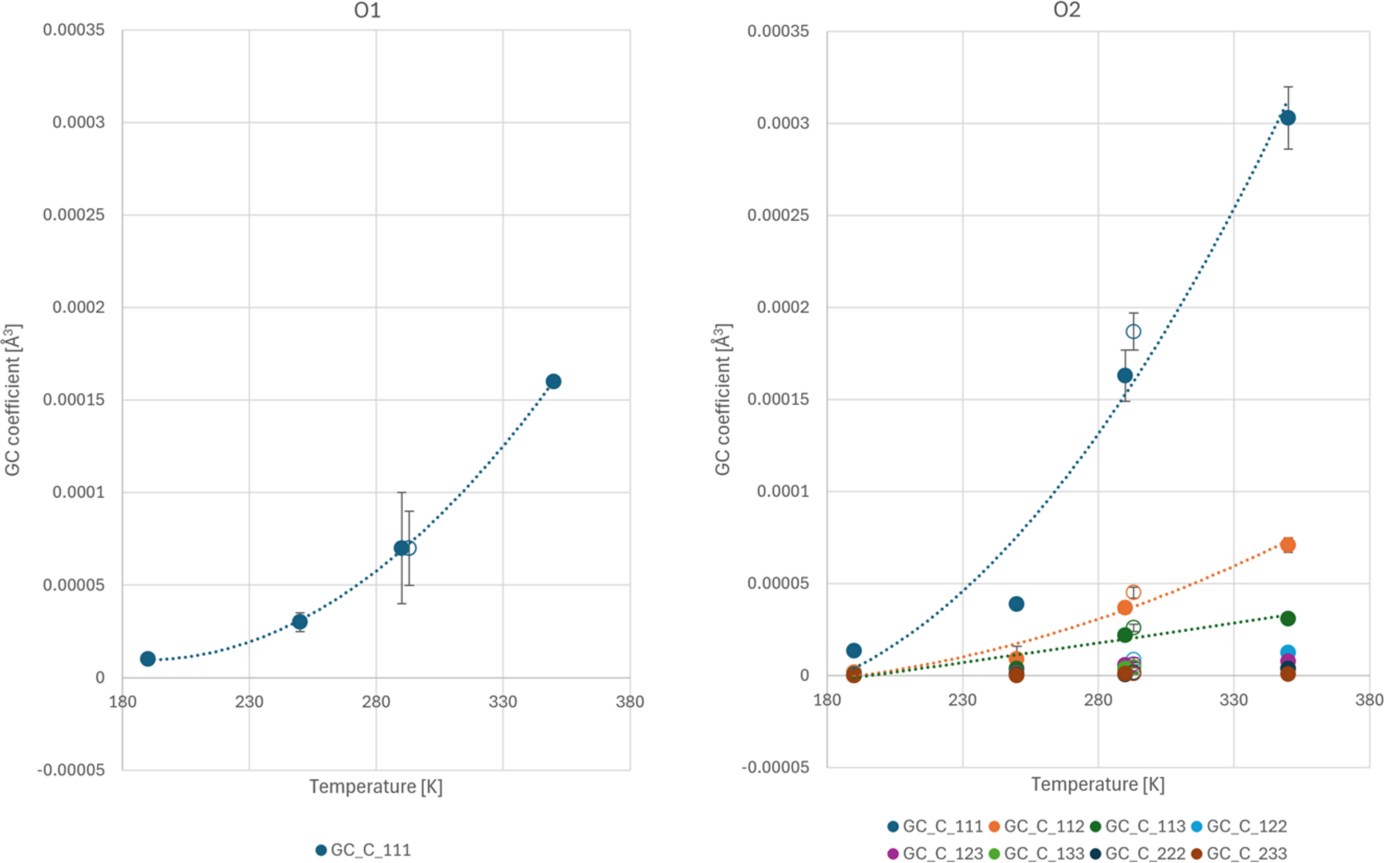
Statistically significant GC coefficients refined for the O1 and O2 carbonyl oxygen atoms in 2°AP-β as a function of temperature. Near-quadratic dependencies are visible. The hollow point represents a result from our former work, coinciding nicely with the current series.

Notably, the statistically significant GC coefficients retain their significance with increasing temperature, showing nice quadratic dependencies, as illustrated in Fig. 9 ▸. Their distributions clearly illustrate the distinct behavior of O1 and O2. In the case of the former, one third-order GC coefficient sufficiently models observed anharmonic effects, whereas oscillations of the O2 carbonyl require a few GC coefficients of both third and fourth order to be fully corrected for. In accordance with the asymmetrical distribution of positive residual density features (Fig. 5 ▸), the third-order GC coefficients are the most pronounced, explaining the majority of observed effects.

### 3.4. Interpretation of the refined models

**Figure 10:**
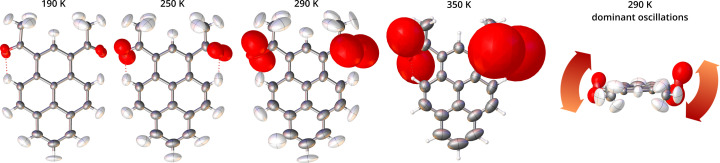
Molecule of 2°AP-β as refined at selected temperatures. Harmonic anisotropic ADPs represented at the 50% probability level. Anharmonic contributions to the total probability distribution function for O1 and O2 are represented as red (peanut-shaped) surfaces and scaled by 1.5 for better visibility. Their long axes indicate the direction of anharmonic oscillations of each atom.

The current implementation of the anharmonic corrections in the GC formalism in *Olex2* allowed us to visualize the additional contribution added to the Gaussian probability distribution represented by the anisotropic displacement ellipsoid. Such contributions, scaled up for better visibility, are presented for the oxygen atoms in 2°AP-β in Fig. 10 ▸.

They can be interpreted as additional elongation of the probability distribution isosurface in the direction of the dominant carbonyl group oscillation (roughly [100]). In other words, the oxygen atoms are oscillating out of the mean position in the direction indicated by the long axis of the displayed shape much further out than would be allowed by a harmonic model. As expected, these vibrations become more pronounced with increased temperature.

**Figure 11:**
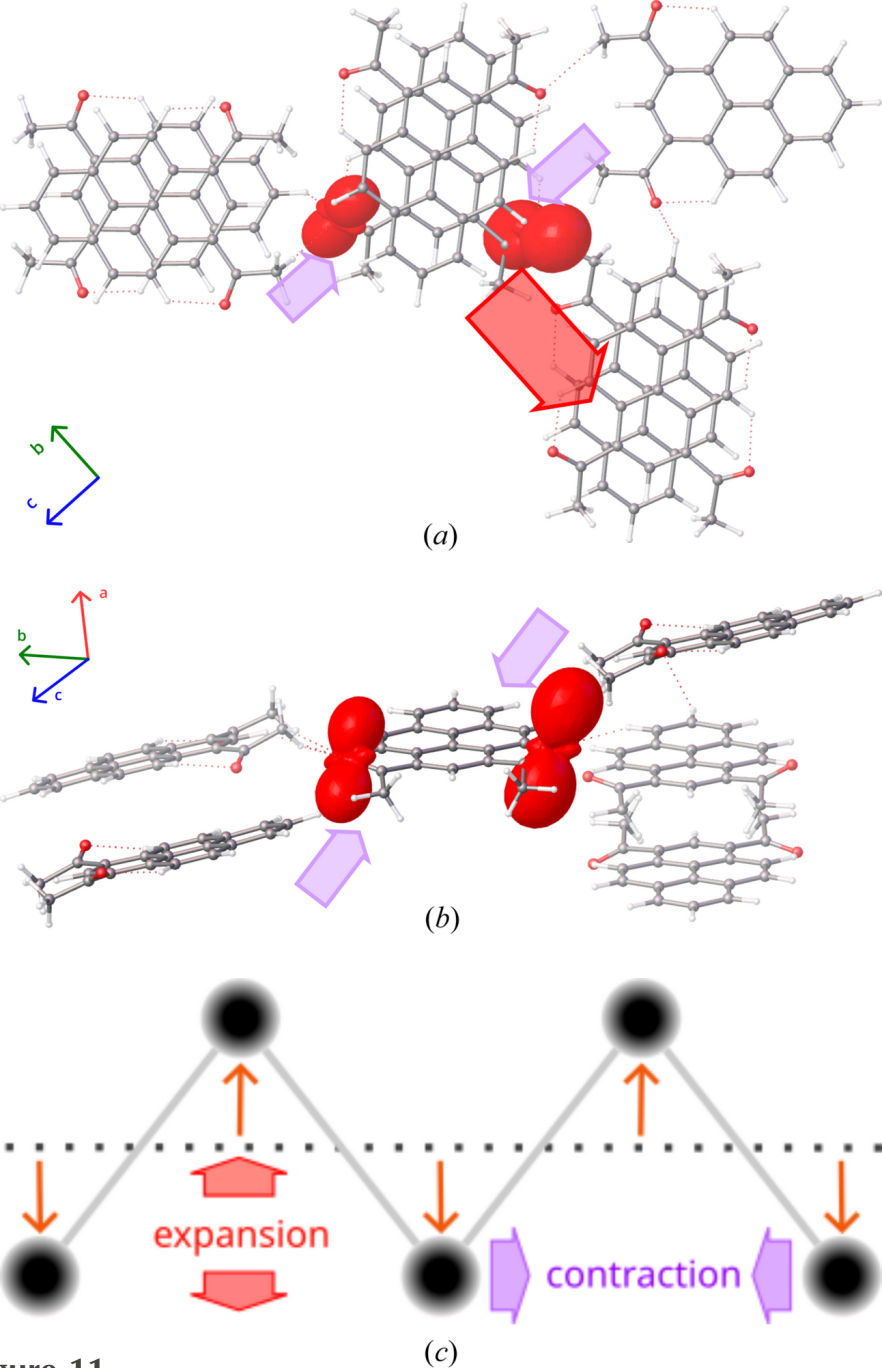
Molecule of 2°AP-β with its closest neighbors in the crystal structure viewed along (*a*) the π⋯π stack and (*b*) from the side, as refined at 290 K. Harmonic anisotropic ADPs represented at the 50% probability level. Anharmonic contributions to the total probability distribution function are represented as red (peanut-shaped) isosurfaces and scaled by 1.5 for better visibility. The arrows indicate the principal axes of the thermal expansion (red – positive, lavender – negative) superimposed on the crystal structure. The arrow widths are scaled by the thermal expansion α_*L*_ coefficients. (*c*) Schematic of the influence of prominent transverse vibrations on a ribbon of objects.

As described in our former work (Zwolenik *et al.*, 2024 ▸), two of the apparently weakest C—H⋯O hydrogen bonds (namely C20—H20b⋯O1 and C18—H18b⋯O2) are collinear with the direction of the NTE eigenvector (approximately [302]). These interactions form a ribbon of molecules, which undulates with the increased oscillation amplitudes while preserving certain H⋯O interatomic distances. The most significant anharmonic motions of oxygen atoms occur in the direction perpendicular to the surface of this molecular band. As a consequence, the material expands in that direction, while contracting along [302], as the C—H⋯O hydrogen bonds ‘tilt’ instead of stretching (Fig. 11 ▸). The mechanism of the enormous NTE of crystalline 2°AP-β can thus be explained based on the presence of significant transverse vibrations in a line (ribbon) of 1,3-diacetylpyrene molecules linked by C—H⋯O interactions, as described by Attfield (2018 ▸).

A few recent studies showed that phenomena such as anisotropic thermal expansion (Juneja *et al.*, 2024 ▸) or certain disorder (Hutereau *et al.*, 2020 ▸) can be linked to pronounced anharmonicity in just one or two specific vibrational modes. In the case of 2°AP-β, a few normal vibrational modes theoretically predicted for 2°AP-β indeed show significant displacements of O1 and O2 atoms in the directions indicated by the refined GC model, synchronous with oscillations of the C—H⋯O connected methyl groups from adjacent molecules so as to preserve the C—H⋯O relations. These are located in the range between 80 and 150 cm^−1^ (Raman- as well as IR-active modes, a list is provided in Section S9 of the supporting information). Visualizations of the two most prominent modes, at 81 and 135 cm^−1^ accordingly, are also provided with the supporting information. They represent the vibrational modes intermediate between soft (lattice) and hard (molecular), with non-negligible contributions from both (Bürgi & Capelli, 2000 ▸). Importantly, these relatively high-frequency modes would not normally be included/refined in the analysis of thermodynamic properties or the material with approaches such as *NoMoRe* (Hoser & Madsen, 2017 ▸; Hoser *et al.*, 2024 ▸).

As these few vibrational modes involve synchronous motions of both oxygen atoms and –CH_3_ groups, the latter would be expected to show some signs of anharmonicity. However, there were no residual density features indicating the need for anharmonic corrections of the methyl carbons. This could be due to the fact that the expected transverse motions of these carbon atoms are convoluted with, for example, rotational motions, and hence too little pronounced to be detectable with the limited resolution of the current X-ray data.

In the absence of the experimental Raman/THz spectra, one way to confirm our interpretation would be to map out the potential energy along the coordinates of selected vibrational modes for structures determined at a series of temperatures, analogous to the work of Juneja *et al.* (2024 ▸), and check the extent to which potential energy wells flatten with temperature. Such theoretical investigations for a range of diacetyl­pyrenes are ongoing and will be the subject of further communications.

## 4. Conclusions

We systematically studied the onset and evolution of the anharmonic effects in crystalline vibrations in the low-symmetry β polymorph of 1,3-diacetylpyrene using single-crystal X-ray diffraction experiments conducted under standard laboratory conditions over a wide temperature range up to 390 K. The diffraction experiments were interpreted by crystal structure refinement. Aspherical atomic scattering factors derived from HAR were used exclusively to account for the static bonding density features. They allowed for unbiased modeling of dynamic anharmonic effects with the Gram–Chalier approximation, as implemented in widely available and easily used *Olex2*.

In extension to our previous work, the onset of substantial anharmonic effects was established to occur at 190 K. This was the lowest temperature at which anharmonic corrections for atomic displacements were necessary to account for residual density features and at which the obtained GC coefficients were statistically significant. The enormous NTE coefficient of crystalline 2°AP-β in the [302] direction, reaching −199 (6) MK^−1^ at around 300 K, has been re-established based on an exact linear fit to a subset of data between 210 and 330 K. It can be explained based on the presence of significant transverse vibrations of oxygen atoms in acetyl substituents within ribbons formed by 1,3-diacetylpyrene molecules linked by ‘rigid’ C—H⋯O hydrogen bonds. The direction of these vibrations was indicated by the anharmonic GC corrections to the atomic probability distributions and corroborated by the presence of likely normal vibrational modes predicted by means of qualitative theoretical DFT calculations at approximately 80 and 135 cm^−1^.

Our results show that the anharmonic effects can be quantified for organic materials based exclusively on light elements, containing larger molecules and displaying quite complex lattice vibrations. Such quantification is possible once the deformations of static scattering density resulting from asphericity of atomic electron densities has been accounted for by aspherical atomic scattering factors provided by modern quantum crystallography approaches such as for instance HAR. While performing such analysis demands collecting highly reliable diffraction intensities, the necessary data resolution is limited by the number-of-data to number-of-parameters ratio and the extent of observed anharmonicity rather than by any arbitrary criterion. As a result, diffraction experiments sufficient for characterization of anharmonic effects can be performed using the current *in house* diffractometer, even at room temperature and only slightly above the standard resolution limit of 0.82 Å (Spek, 2020 ▸). This opens up the possibility of such studies for a much wider range of organic materials that, until now, were not considered due to their limited X-ray scattering power. In particular, our research shows that anharmonic vibrations can be detected even for photo-luminescent samples that are non-viable for Raman spectroscopic studies.

Although the GC expansion parameters obtained in the course of crystallographic study do not transcribe into corrections which could directly help to predict or interpret IR/Raman spectra, the total probability density distributions for atoms obtained from our experiments can still be used to validate the outcomes of computational approaches (molecular dynamics, quantum-chemical calculations) used in predicting material properties.

## 5. Related literature

The following reference is cited in the supporting information: Allen & Bruno (2010) ▸.

## Supplementary Material

Crystal structure: contains datablock(s) 110K, 150K, 190K, 250K, 290K, 350K. DOI: 10.1107/S2052252524010443/pen5002sup1.cif

Animation of lattice vibration at 135 cm-1. DOI: 10.1107/S2052252524010443/pen5002sup2.gif

Animation of lattice vibration at 116 cm-1. DOI: 10.1107/S2052252524010443/pen5002sup3.gif

Experimental details, structure deposition numbers and supplementary tables and figures. DOI: 10.1107/S2052252524010443/pen5002sup4.pdf

Original XRD images and associated metadata for diffraction experiments in the 110-350 K temperature range : https://doi.org/10.18150/SWDNTW

Aspherical atomic scattering factors generated in the course of Hirshfeld atom refinements for structures determined at 110, 150, 190, 250, 290 and 350K: https://doi.org/10.18150/UDX1VQ

CCDC references: 2379227, 2379228, 2379229, 2379230, 2379231, 2379232

## Figures and Tables

**Table 1:** Selected crystallographic data and structure refinement statistics for 2°AP-β (C_20_H_14_O_2_, *M*_r_ = 286.33 g mol^−1^)

Data collection						
Temperature (K)	110	150	190	250	290	350
Crystal system	Monoclinic	Monoclinic	Monoclinic	Monoclinic	Monoclinic	Monoclinic
Space group	*P*2_1_/*c*	*P*2_1_/*c*	*P*2_1_/*c*	*P*2_1_/*c*	*P*2_1_/*c*	*P*2_1_/*c*
*Z*, *Z*′	4, 1	4, 1	4, 1	4, 1	4, 1	4, 1
*a* (Å)	7.14940 (18)	7.17156 (18)	7.1945 ▸(2)	7.2211 (2)	7.2277 (6)	7.2522 (5)
*b* (Å)	10.9139 (3)	10.9455 (3)	10.9948 (3)	11.0779 (3)	11.2480 (9)	11.4389 (8)
*c* (Å)	17.7592 (5)	17.7694 (5)	17.7698 (5)	17.7769 (5)	17.7562 (14)	17.7050 (13)
β (°)	102.584 (3)	102.742 (3)	102.926 (3)	103.161 (3)	103.578 (8)	104.385 (7)
*V* (Å^3^)	1352.42 (6)	1360.48 (6)	1370.01 (7)	1384.70 (7)	1403.2 (2)	1422.71 (18)
ρ_calc_ (g cm^−3^)	1.406	1.398	1.388	1.373	1.355	1.337
μ (mm^−1^)	0.714	0.710	0.705	0.698	0.689	0.679
*F*(000)	601.96	601.96	601.96	601.96	601.96	601.96
Wavelength (Å)	1.54184	1.54184	1.54184	1.54184	1.54184	1.54184
Reflections collected	20709	20905	9160	9298	8617	9254
*R*_int_	0.0403	0.0417	0.0287	0.0248	0.0340	0.0309
Resolution (Å)	0.7896	0.7894	0.7845	0.7849	0.7849	0.7845
Completeness (%)	0.9992	0.9992	1.0000	0.9968	0.9827	0.9852

Data refinement						
	HAR/NoSpherA2					
Data	2847	2867	2945	2964	2945	3002
Restraints	0	0	0	0	0	0
Parameters	325	325	325	325	325	255
*R*_1_ index [*I* > 2σ(*I*)]	0.023	0.025	0.023	0.029	0.048	0.059
*wR*_2_ index [*I* > 2σ(*I*)]	0.054	0.049	0.057	0.067	0.113	0.167
*R*_1_ index (all data)	0.029	0.032	0.029	0.036	0.090	0.095
*wR*_2_ index (all data)	0.057	0.052	0.060	0.071	0.167	0.209
Max diff. peak [e Å^−3^]	0.130	0.121	0.115	0.204	0.327	0.382
Min diff. hole [e Å^−3^]	−0.113	−0.122	−0.111	−0.166	−0.316	−0.257

	HAR + anh/NoSpherA2					
Parameters	–	–	375	375	375	305
*R*_1_ index [*I* > 2σ(*I*)]	–	–	0.022	0.025	0.037	0.042
*wR*_2_ index [*I* > 2σ(*I*)]	–	–	0.053	0.052	0.077	0.104
*R*_1_ index (all data)	–	–	0.028	0.032	0.079	0.079
*wR*_2_ index (all data)	–	–	0.055	0.055	0.120	0.135
Max diff. peak (e Å^−3^)	–	–	0.095	0.107	0.221	0.150
Min diff. hole (e Å^−3^)	–	–	−0.100	−0.120	−0.266	−0.197

**Table 2:** Thermal expansion α (MK^−1^), based on experimental unit-cell constants and calculated using the *PASCal* server (Cliffe & Goodwin, 2012 ▸)

	Thermal expansion			
		Direction		
Main axis	Value (MK^−1^)	*a*	*b*	*c*
X1	−199 (6)	0.8733	0.0000	0.4871
X2	102 (4)	0.9715	0.0000	−0.2370
X3	409 (8)	0.0000	1.0000	0.0000
