# Supplementary figures and images for: Tracking anharmonic oscillations in the structure of β-1,3-diacetylpyrene
Source: IUCrJ. 2025 Jan 1;12(Pt 1):23–35. doi: 10.1107/S2052252524010443 (PMC11707697; doi:10.1107/S2052252524010443)

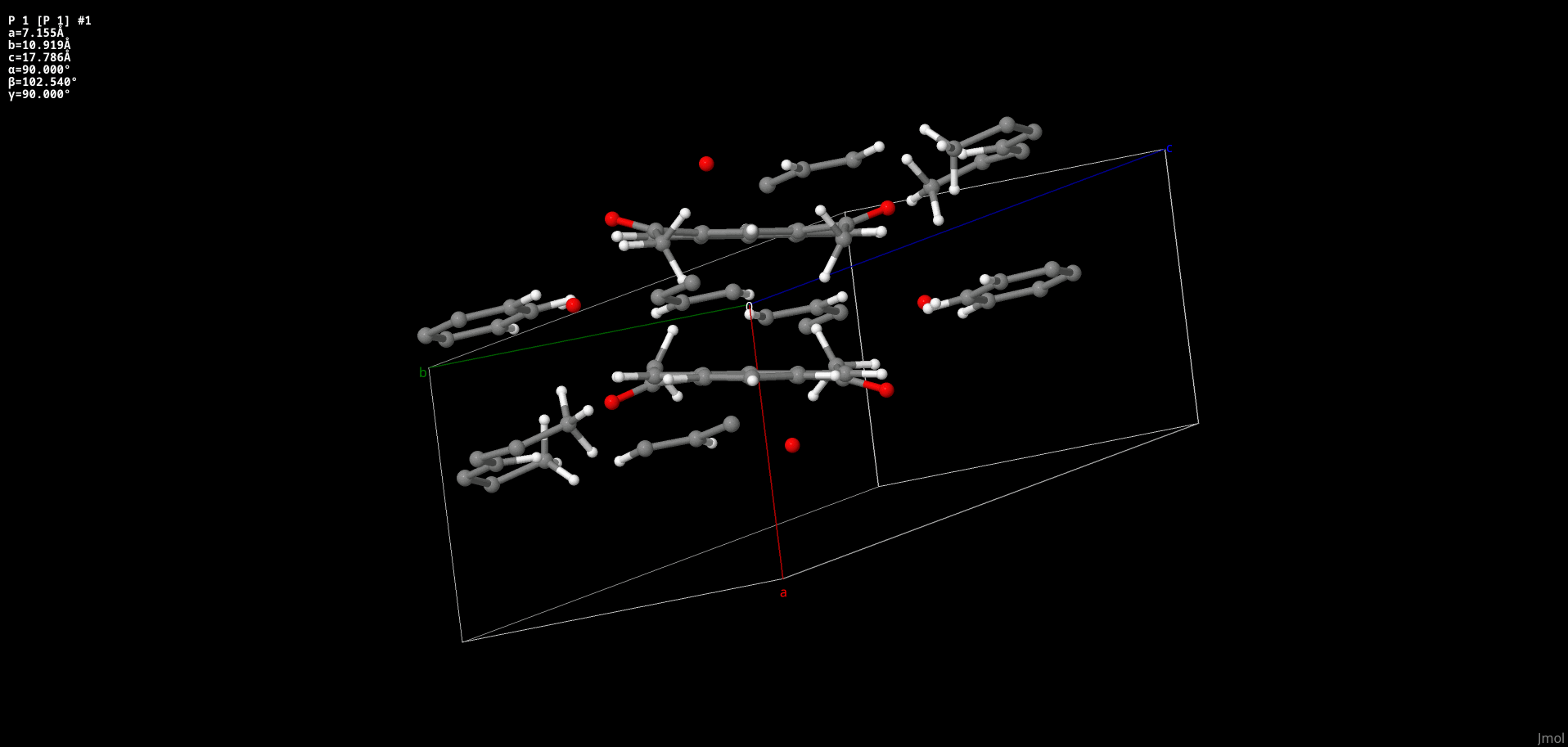

Supplement: Supplementary file 2 [file m-12-00023-sup2.gif]

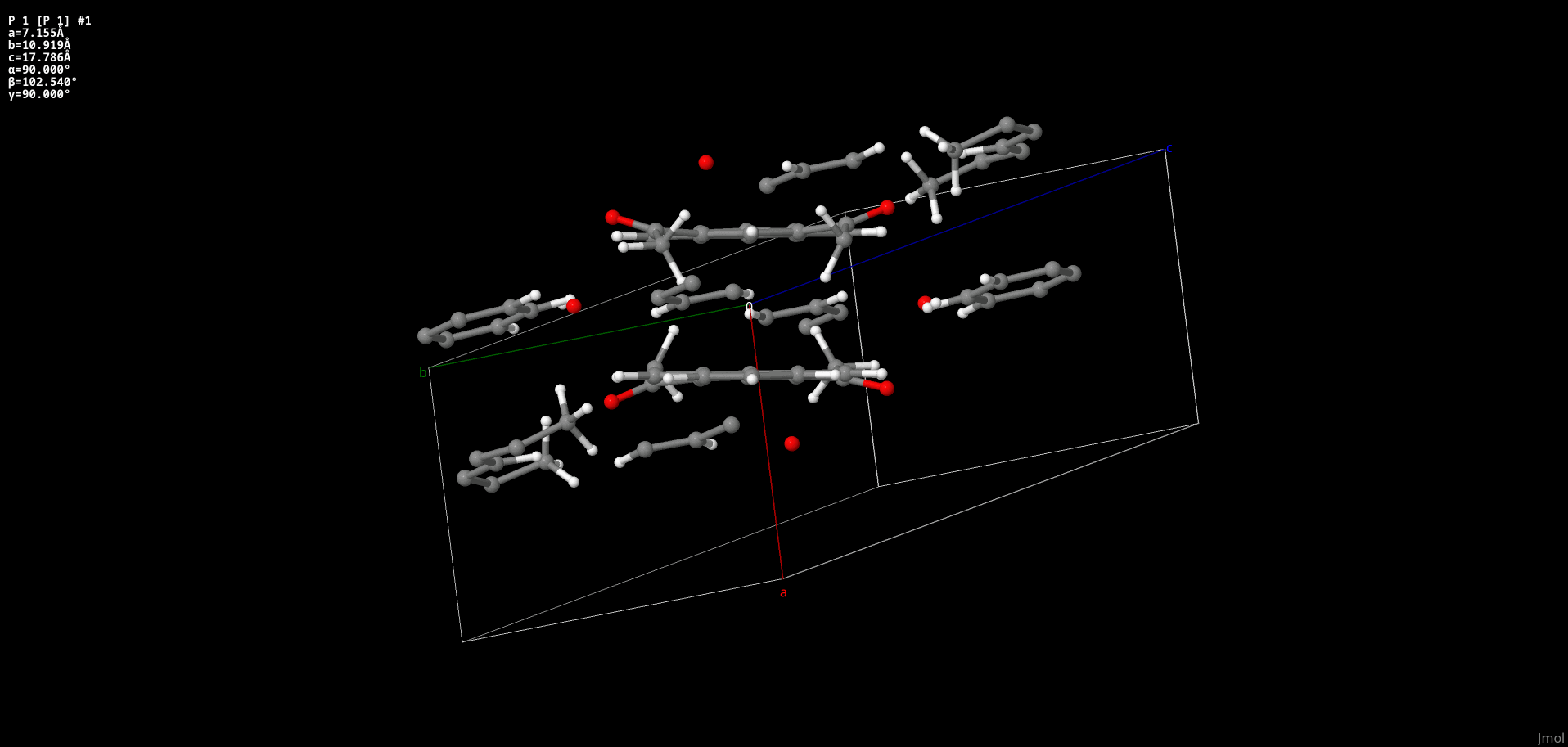

Supplement: Supplementary file 3 [file m-12-00023-sup3.gif]
