# Supplementary material for: Tracking anharmonic oscillations in the structure of β-1,3-diacetylpyrene
Source: IUCrJ. 2025 Jan 1;12(Pt 1):23–35. doi: 10.1107/S2052252524010443 (PMC11707697; doi:10.1107/S2052252524010443)
Supplement: Supplementary file 4 [file m-12-00023-sup4.pdf]

IUCrJ

Volume 12 (2025)

Supporting information for article:

Tracking anharmonic oscillations in the structure of β-1,3-diacetylpyrene

A. Zwolenik and A. Makal

Tracking anharmonic oscillations in the structure of β 1,3-diacetylpyrene

Aleksandra Zwolenik and Anna Makal
contact: *am.makal@uw.edu.pl*

October 22, 2024

Contents

S1 Supporting materials **S2**

S1 Data and structures availability S2
S2 Final crystallographic tables S3
S3 Unit cell parameters S4
S4 Test against the Kuhs' rule S6
S5 Fractal dimension plots S7
S6 Final refined coefficients of the Gram-Charlier anharmonicity correction S8
S7 C-H distances S12
S8 Intermolecular interactions energies of 2°AP-β S13
S9 The lowest-frequency lattice vibration modes for 2°AP-β S14
S10 Differential Scanning Calorimetry S15

S2 Bibliography **S16**

Chapter S1

Supporting materials

S1 Data and structures availability

Structures determined for the purpose of this paper were deposited as individual entries within Cambridge Structural Database. [Groom et al., 2016] Exact deposition number for each structure have been presented in Table S1.1.

Table S1.1: *CCDC deposition numbers of crystal structures determined for the purpose of these studies, sorted by temperature of the experiment.*

T	CCDC deposition number
110K	2379228
150K	2379227
190K	2379232
250K	2379231
290K	2379229
350K	2379230

Additionally, original X-ray diffraction images and associated data are available online using the following doi: `https://doi.org/10.18150/SWDNTW` from the Repository for Open Data (RepOD `https://repod.icm.edu.pl/`, Repository for Open Data, Interdisciplinary Centre for Mathematical and Computational Modelling, University of Warsaw, Warsaw, Poland). The aspherical atomic scattering factors generated in the course of Hirshfeld Atom Refinements are also available online from RepOD using the following doi: `https://doi.org/10.18150/UDX1VQ`

S2 Final crystallographic tables

Table S2.1: *Crystallographic data and performed refinements.*

Temperature [K]	110	150	190	250	290	350
Formula	$C_{20}H_{14}O_2$	$C_{20}H_{14}O_2$	$C_{20}H_{14}O_2$	$C_{20}H_{14}O_2$	$C_{20}H_{14}O_2$	$C_{20}H_{14}O_2$
Weight $[\frac{g}{mol}]$	286.333	286.333	286.333	286.333	286.333	286.333
Crystal system	monoclinic	monoclinic	monoclinic	monoclinic	monoclinic	monoclinic
Space group	$P2_1/c$	$P2_1/c$	$P2_1/c$	$P2_1/c$	$P2_1/c$	$P2_1/c$
a [Å]	7.14940(18)	7.17156(18)	7.1945(2)	7.2211(2)	7.2277(6)	7.2522(5)
b [Å]	10.9139(3)	10.9455(3)	10.9948(3)	11.0779(3)	11.2480(9)	11.4389(8)
c [Å]	17.7592(5)	17.7694(5)	17.7698(5)	17.7769(5)	17.7562(14)	17.7050(13)
α [°]	90	90	90	90	90	90
β [°]	102.584(3)	102.742(3)	102.926(3)	103.161(3)	103.578(8)	104.385(7)
γ [°]	90	90	90	90	90	90
Volume [Å^3]	1352.42(6)	1360.48(6)	1370.01(7)	1384.70(7)	1403.2(2)	1422.71(18)
Z, Z'	4, 1	4, 1	4, 1	4, 1	4, 1	4, 1
ρ_{calc} $[\frac{g}{cm^3}]$	1.406	1.398	1.388	1.373	1.355	1.337
μ $[mm^{-1}]$	0.714	0.710	0.705	0.698	0.689	0.679
F(000)	601.956	601.956	601.956	601.956	601.956	601.956
Max size $[mm^3]$	0.175	0.175	0.18	0.183	0.183	0.183
Mid size $[mm^3]$	0.135	0.135	0.13	0.129	0.129	0.129
Min size $[mm^3]$	0.093	0.093	0.11	0.107	0.107	0.107
X-Ray source	Cu Kα	Cu Kα	Cu Kα	Cu Kα	Cu Kα	Cu Kα
Wavelength [Å]	1.54184	1.54184	1.54184	1.54184	1.54184	1.54184
2θ range	9.58-155.02	9.56-155.16	9.52-158.66	9.48-158.34	9.38-158.36	9.3-158.66
Reflections collected	20709	20905	9160	9298	8617	9254
R_{int}	0.0403	0.0417	0.0287	0.0248	0.0340	0.0309
Resolution [Å]	0.7896	0.7894	0.7845	0.7849	0.7849	0.7845
Completeness [%]	0.9992	0.9992	1.0000	0.9968	0.9827	0.9852
	HAR / NoSpherA2					
Data	2847	2867	2945	2964	2945	3002
Restraints	0	0	0	0	0	0
Parameters	325	325	325	325	325	255
R_1 index ($I > 2\sigma(I)$)	0.0229	0.0251	0.0234	0.0288	0.0481	0.0593
wR_2 index ($I > 2\sigma(I)$)	0.0543	0.0499	0.0576	0.0674	0.1125	0.1671
R_1 index (all data)	0.0287	0.0318	0.0289	0.0364	0.0902	0.0954
wR_2 index (all data)	0.0567	0.0522	0.0601	0.0710	0.1665	0.2087
Max diff. peak [eÅ^{-3}]	0.1301	0.1206	0.1151	0.2040	0.3274	0.3816
Max diff. hole [eÅ^{-3}]	-0.1126	-0.1223	-0.1113	-0.1657	-0.3161	-0.2574
	HAR + anh / NoSpherA2					
Data	-	-	2945	2964	2945	3002
Restraints	-	-	0	0	0	0
Parameters	-	-	375	375	375	305
R_1 index ($I > 2\sigma(I)$)	-	-	0.0221	0.0245	0.0373	0.0418
wR_2 index ($I > 2\sigma(I)$)	-	-	0.0532	0.0524	0.0770	0.1036
R_1 index (all data)	-	-	0.0277	0.0319	0.0794	0.0785
wR_2 index (all data)	-	-	0.0555	0.0552	0.1200	0.1345
Max diff. peak [eÅ^{-3}]	-	-	0.0950	0.1070	0.2210	0.1500
Max diff. hole [eÅ^{-3}]	-	-	-0.1003	-0.1198	-0.2566	-0.1970

S3 Unit cell parameters

Table S3.1: *Unit cell parameters of structures studied in multi-temperature diffraction measurements.*

T [K]	a [Å]	b [Å]	c [Å]	β [°]	V [Å^3]	R_{int}
110-230 K, Cu Kα						
110	7.14940(18)	10.9139(3)	17.7592(5)	102.584(3)	1352.42(6)	0.0405
120	7.15390(18)	10.9223(3)	17.7632(5)	102.606(3)	1354.50(6)	0.0392
130	7.15997(17)	10.9309(3)	17.7658(5)	102.654(3)	1356.66(6)	0.0400
140	7.16439(17)	10.9383(3)	17.7682(5)	102.692(2)	1358.41(6)	0.0418
150	7.17156(18)	10.9455(3)	17.7694(5)	102.742(3)	1360.48(6)	0.0417
160	7.17601(18)	10.9558(3)	17.7734(5)	102.765(3)	1362.79(6)	0.0407
170	7.18144(18)	10.9644(3)	17.7758(5)	102.791(3)	1364.94(6)	0.0437
180	7.18713(18)	10.9759(3)	17.7771(5)	102.835(3)	1367.31(6)	0.0405
190	7.19279(15)	10.9883(2)	17.7785(5)	102.878(2)	1369.8(5)	0.0411
200	7.19749(16)	10.9995(3)	17.7789(5)	102.915(2)	1371.92(6)	0.0403
210	7.20349(17)	11.0121(3)	17.7792(5)	102.950(2)	1374.47(6)	0.0409
220	7.20794(19)	11.0291(3)	17.7743(5)	102.989(3)	1376.85(6)	0.0407
230	7.21373(18)	11.0425(3)	17.7733(5)	103.013(2)	1379.42(6)	0.0443
190-350 K, Cu Kα						
190	7.1938(3)	10.9961(4)	17.7693(4)	102.924(3)	1370.02(8)	0.0290
210	7.2013(2)	11.0129(4)	17.7647(6)	102.984(3)	1372.85(9)	0.0254
230	7.2130(3)	11.0409(5)	17.7771(7)	103.058(3)	1379.12(9)	0.0236
250	7.2247(3)	11.0782(4)	17.7692(6)	103.181(3)	1384.71(9)	0.0249
270	7.2308(3)	11.1336(5)	17.7550(6)	103.324(3)	1390.89(10)	0.0243
290	7.2274(7)	11.2494(9)	17.7506(9)	103.520(6)	1403.19(19)	0.0342
310	7.2422(4)	11.3330(6)	17.7296(8)	103.947(4)	1412.28(12)	0.0328
330	7.2449(4)	11.4006(7)	17.6976(8)	104.260(4)	1416.72(13)	0.0281
350	7.2548(6)	11.4451(10)	17.6934(11)	104.441(7)	1422.7(2)	0.0309

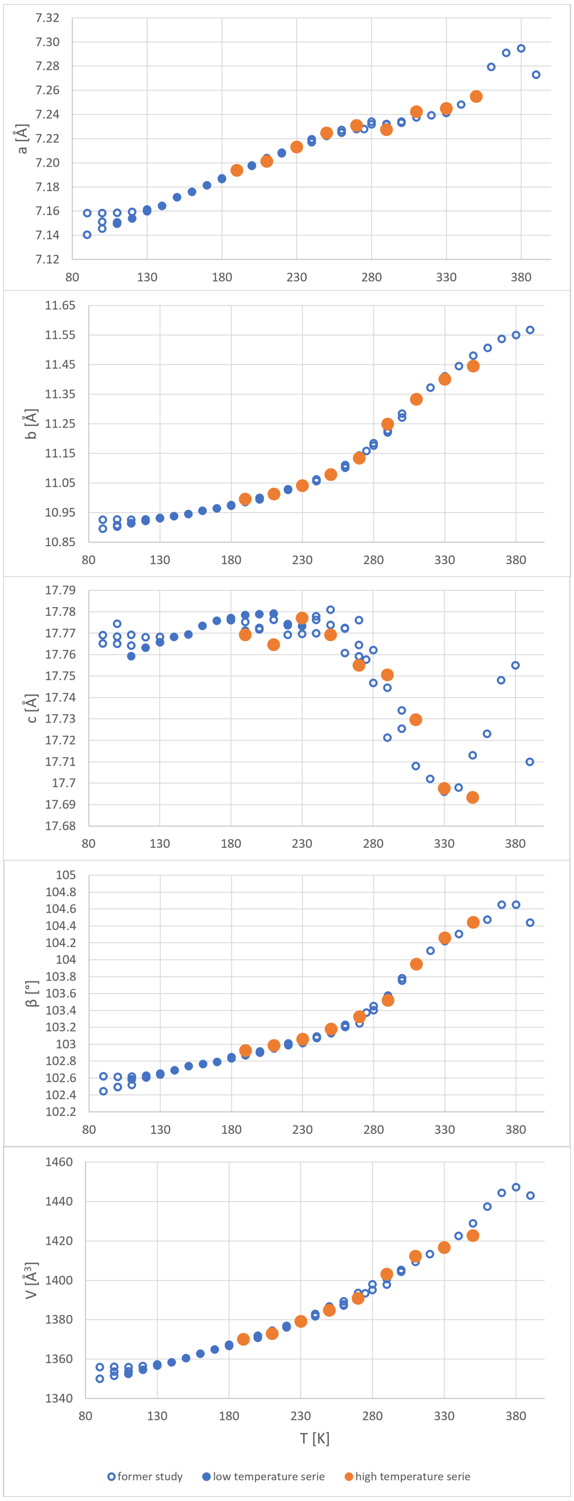

Figure S3.1: *Evolution of the unit cell parameters with temperature. Hollow points illustrate data from our former work [Zwolenik et al., 2024], large orange points refer to data for which crystal structure has been refined in the current work.*

S4 Test against the Kuhs' rule

Suitability of the diffraction data for refinement including Gram-Charlier formalism of anharmonic corrections for a given atom was ascertained using a formula proposed by Kuhs [Kuhs, 1988] from average harmonic u^2 mean-square displacement parameters of that atom

$$h_n = n^{\frac{1}{2}}(2\pi)^{-\frac{3}{2}}(2ln2)^{\frac{1}{2}}\left\langle u^2\right\rangle^{-\frac{1}{2}} \qquad \text{(S1.1)}$$

where n indicates the order of Gram-Charlier expansion and h_n is the minimal required resolution in Å^{-1}.

Table S4.1: *Minimal resolution required to perform given refinement of anharmonic corrections ascertained using Kuhs formula[Kuhs, 1988] (Equation S1.1).*

	Minimal required resolution [Å^{-1}]						
	110K	150K	190K	250K	290K	350K	293K
O1, 3rd order	0.66	0.75	0.85	1.00	1.16	1.33	1.14
O2, 3rd order	0.74	0.85	0.97	1.17	1.41	1.86	1.42
O1, 4th order	0.57	0.65	0.73	0.87	1.01	1.16	0.99
O2, 4th order	0.64	0.74	0.84	1.01	1.22	1.61	1.23

S5 Fractal dimension plots

Fractal dimension plots [Meindl and Henn, 2008] for structures at 190 K (Figure S5.1a) and 290K (Figure S5.1b) refined without or with anharmonic atomic displacement corrections in GC formalism for models using either spherical atomic form-factors or HAR refinement. Ideal parabolic shapes suggest a random distribution of residual density, i.e. a structural model well fitted to experimental data. Both aspherical atomic form factors from HAR and Gram-Charlier coefficients are necessary to improve these plots as they address different systematic effects: the former improve the parabolic shape while the latter account for the asymmetric broadening on the positive side. As expected, the contribution from aspherical atomic form factors (HAR) is dominant at lower temperature.

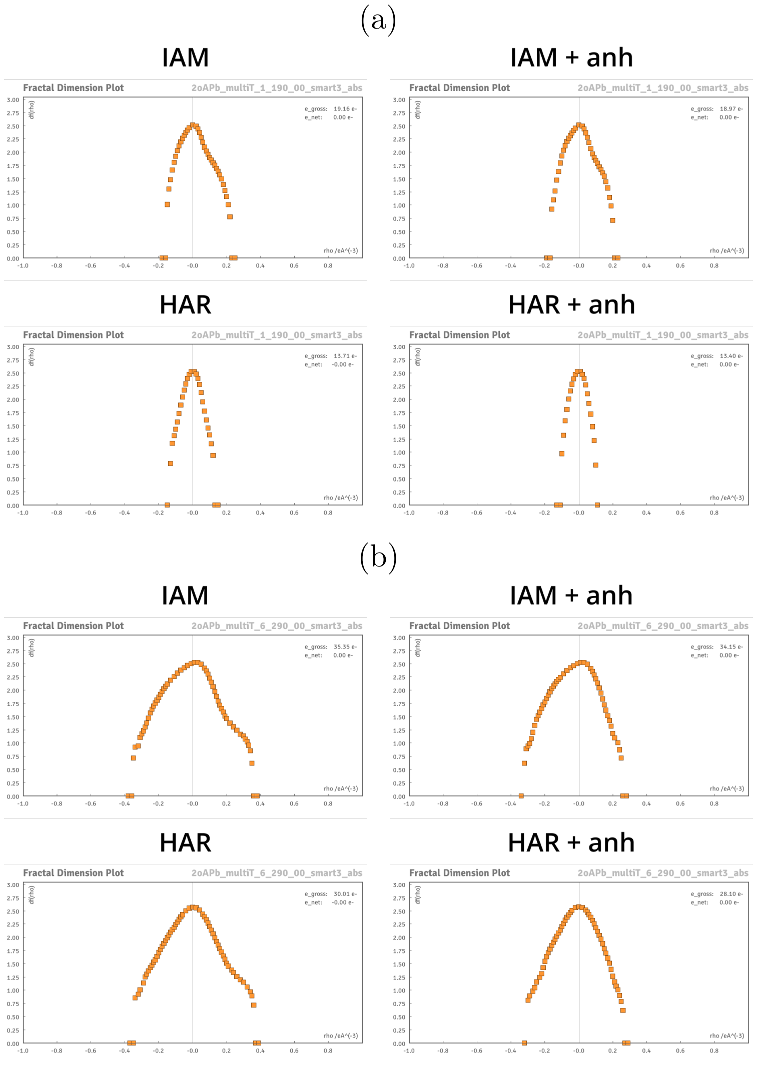

Figure S5.1: *Fractal dimension plots [Meindl and Henn, 2008] for structures at 190 K (a) and 290 K (b) refined without (left) or with (right) anharmonic atomic displacement corrections in GC formalism for models using spherical atomic form-factors (top) and HAR refinement (bottom).*

S6 Final refined coefficients of the Gram-Charlier anharmonicity correction

Table S6.1: *A listing of refined Gramm-Charlier parameters describing correction for anharmonicity in thermal motions of O1 and O2 atoms at 190K. Statistically significant coefficients are represented in bold.*

	O1	O2
3rd order		
GC_C_111	**0.000010(3)**	**0.0000137(14)**
GC_C_112	0.0000006(9)	**-0.0000019(4)**
GC_C_113	-0.0000002(7)	0.0000008(3)
GC_C_122	0.0000004(5)	0.0000003(2)
GC_C_123	-0.0000000(2)	**-0.00000042(11)**
GC_C_133	-0.0000000(2)	0.00000019(11)
GC_C_222	0.0000004(5)	0.0000001(2)
GC_C_223	0.00000022(18)	-0.00000007(8)
GC_C_233	0.00000000(12)	0.00000004(6)
GC_C_333	-0.00000005(13)	0.00000012(6)
4th order		
GC_D_1111	0.0000003(4)	0.00000027(19)
GC_D_1112	-0.00000003(12)	-0.00000011(5)
GC_D_1113	0.00000001(8)	0.00000007(4)
GC_D_1122	0.00000002(5)	0.00000005(2)
GC_D_1123	-0.00000000(3)	-0.000000033(12)
GC_D_1133	-0.00000001(2)	0.000000025(12)
GC_D_1222	0.00000002(4)	-0.000000007(16)
GC_D_1223	0.000000018(14)	0.000000013(6)
GC_D_1233	-0.000000003(9)	-0.000000009(5)
GC_D_1333	-0.000000001(11)	0.000000010(5)
GC_D_2222	0.00000001(4)	-0.000000023(17)
GC_D_2223	0.000000015(14)	0.000000004(6)
GC_D_2233	0.000000002(7)	0.000000003(3)
GC_D_2333	-0.000000002(6)	-0.000000004(3)
GC_D_3333	-0.000000000(8)	0.000000005(4)

Table S6.2: *A listing of refined Gramm-Charlier parameters describing correction for anharmonicity in thermal motions of O1 and O2 atoms at 250K. Statistically significant coefficients are represented in bold.*

	O1	O2
	3rd order	
GC_C_111	**0.000030(5)**	**0.000039(3)**
GC_C_112	-0.0000016(16)	**0.0000090(7)**
GC_C_113	-0.0000007(11)	**0.0000039(6)**
GC_C_122	-0.0000001(8)	**0.0000009(3)**
GC_C_123	0.0000000(4)	**0.00000123(19)**
GC_C_133	0.0000002(4)	**0.00000090(18)**
GC_C_222	-0.0000010(7)	-0.0000005(3)
GC_C_223	0.0000002(3)	-0.00000020(13)
GC_C_233	0.00000007(18)	0.00000006(9)
GC_C_333	0.00000002(19)	0.00000011(9)
	4th order	
GC_D_1111	0.0000015(8)	**0.0000012(4)**
GC_D_1112	-0.0000000(2)	**0.00000041(10)**
GC_D_1113	-0.00000003(16)	-0.00000001(8)
GC_D_1122	0.00000007(9)	0.00000009(4)
GC_D_1123	-0.00000000(5)	0.00000001(2)
GC_D_1133	0.00000001(4)	0.00000001(2)
GC_D_1222	-0.00000001(6)	0.00000001(3)
GC_D_1223	0.00000001(2)	0.000000001(11)
GC_D_1233	0.000000007(16)	0.000000012(8)
GC_D_1333	-0.000000001(17)	0.000000003(8)
GC_D_2222	0.00000006(6)	0.00000001(2)
GC_D_2223	-0.00000001(2)	-0.000000011(10)
GC_D_2233	-0.000000003(11)	0.000000005(5)
GC_D_2333	0.000000004(9)	0.000000003(4)
GC_D_3333	-0.000000005(11)	0.000000004(5)

Table S6.3: *A listing of refined Gramm-Charlier parameters describing correction for anharmonicity in thermal motions of O1 and O2 atoms at 290K. Statistically significant coefficients are represented in bold.*

	O1	O2
3rd order		
GC_C_111	**-0.00007(3)**	**-0.000163(14)**
GC_C_112	-0.000007(7)	**0.000037(3)**
GC_C_113	0.000001(5)	**-0.000022(3)**
GC_C_122	-0.000001(3)	**-0.0000056(13)**
GC_C_123	-0.0000002(17)	**0.0000057(8)**
GC_C_133	-0.0000002(16)	**-0.0000039(8)**
GC_C_222	-0.0000027(18)	-0.0000006(8)
GC_C_223	-0.0000008(10)	-0.0000011(5)
GC_C_233	0.0000001(7)	0.0000011(4)
GC_C_333	0.0000001(8)	-0.0000003(4)
4th order		
GC_D_1111	0.000003(4)	0.000004(2)
GC_D_1112	0.0000002(11)	**-0.0000021(5)**
GC_D_1113	-0.0000003(8)	-0.0000001(4)
GC_D_1122	0.0000000(4)	0.00000050(19)
GC_D_1123	-0.0000000(2)	-0.00000018(12)
GC_D_1133	0.0000000(2)	0.00000000(11)
GC_D_1222	0.0000002(2)	-0.00000004(9)
GC_D_1223	0.00000005(10)	0.00000006(5)
GC_D_1233	-0.00000000(8)	-0.00000004(4)
GC_D_1333	-0.00000002(9)	0.00000005(4)
GC_D_2222	-0.00000025(14)	**-0.00000037(6)**
GC_D_2223	-0.00000002(7)	**-0.00000010(3)**
GC_D_2233	-0.00000001(5)	-0.00000001(2)
GC_D_2333	0.00000000(4)	-0.00000001(2)
GC_D_3333	-0.00000002(5)	0.00000005(2)

Table S6.4: *A listing of refined Gramm-Charlier parameters describing correction for anharmonicity in thermal motions of O1 and O2 atoms at 350K. Statistically significant coefficients are represented in bold.*

	O1	O2
	3rd order	
GC_C_111	**-0.00016(3)**	**-0.000303(17)**
GC_C_112	0.000015(9)	**-0.000071(4)**
GC_C_113	0.000004(7)	**-0.000031(3)**
GC_C_122	-0.000000(3)	**-0.0000125(16)**
GC_C_123	-0.000001(2)	**-0.0000077(10)**
GC_C_133	-0.000001(2)	**-0.0000038(10)**
GC_C_222	0.000002(2)	**-0.0000038(10)**
GC_C_223	**-0.0000055(11)**	-0.0000009(5)
GC_C_233	-0.0000006(8)	-0.0000008(4)
GC_C_333	-0.0000000(9)	-0.0000001(5)
	4th order	
GC_D_1111	0.000004(6)	**-0.000048(3)**
GC_D_1112	-0.0000009(15)	**-0.0000080(7)**
GC_D_1113	-0.0000003(11)	**-0.0000092(6)**
GC_D_1122	0.0000000(5)	**-0.0000017(3)**
GC_D_1123	0.0000001(3)	**-0.00000143(16)**
GC_D_1133	0.0000001(3)	**-0.00000139(15)**
GC_D_1222	-0.0000001(2)	-0.00000027(11)
GC_D_1223	0.00000001(13)	**-0.00000023(6)**
GC_D_1233	0.00000003(10)	-0.00000012(5)
GC_D_1333	-0.00000002(10)	-0.00000009(5)
GC_D_2222	-0.00000001(18)	-0.00000019(8)
GC_D_2223	-0.00000005(9)	0.00000004(4)
GC_D_2233	-0.00000007(6)	0.00000000(3)
GC_D_2333	0.00000002(5)	0.00000004(2)
GC_D_3333	-0.00000002(6)	0.00000005(3)

S7 C-H distances

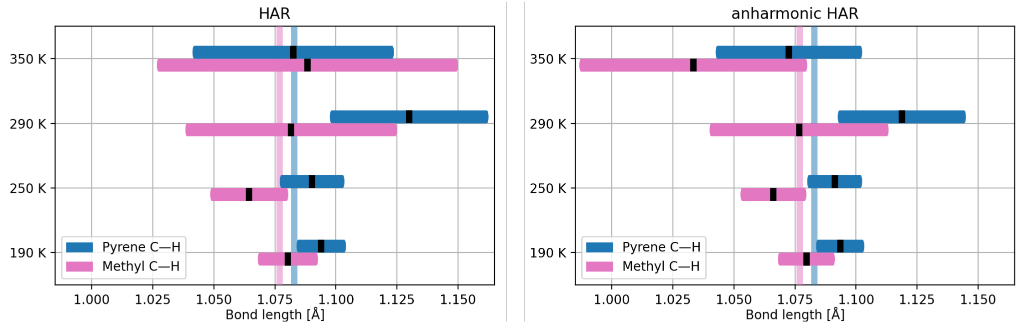

Figure S7.1: *Average C–H bond lengths in pyrene (blue) and methyl (pink) moieties determined by HAR and anharmonic refinements against selected datasets. Theoretical average neutron distances were drawn as vertical lines for comparison[Allen and Bruno, 2010].*

Table S7.1: *C–H bond lengths in pyrene and methyl moieties determined by HAR and anharmonic refinements against selected datasets.*

		Bond length [Å]							
		HAR				anharmonic HAR			
Pyrene C-H		190K	250K	290K	350K	190K	250K	290K	350K
C3	H3	1.084(10)	1.084(12)	1.11(3)	1.08(3)	1.086(9)	1.087(10)	1.12(2)	1.05(2)
C4	H4	1.112(10)	1.088(14)	1.15(3)	1.10(4)	1.113(10)	1.09(12)	1.12(3)	1.07(3)
C6	H6	1.100(12)	1.094(17)	1.07(4)	1.12(5)	1.101(12)	1.093(14)	1.08(4)	1.13(4)
C7	H7	1.084(9)	1.070(13)	1.14(3)	1.05(5)	1.082(9)	1.064(11)	1.12(2)	1.04(4)
C8	H8	1.105(11)	1.109(14)	1.13(3)	1.05(5)	1.103(10)	1.109(12)	1.12(3)	1.05(3)
C10	H10	1.092(10)	1.093(12)	1.12(3)	1.05(4)	1.091(10)	1.097(10)	1.13(2)	1.06(3)
C11	H11	1.095(10)	1.094(13)	1.18(4)	1.13(4)	1.091(10)	1.095(11)	1.12(3)	1.09(3)
C14	H14	1.080(9)	1.090(11)	1.14(3)	1.08(3)	1.081(9)	1.095(10)	1.14(2)	1.09(2)
Average		1.094(10)	1.090(13)	1.13(3)	1.08(4)	1.094(10)	1.091(11)	1.12(3)	1.07(3)
Methyl C-H		190K	250K	290K	350K	190K	250K	290K	350K
C18	H18a	1.099(12)	1.084(17)	1.07(4)	1.06(6)	1.093(12)	1.075(15)	1.05(4)	0.97(4)
C18	H18b	1.072(12)	1.051(15)	1.10(4)	1.05(5)	1.072(11)	1.059(12)	1.11(3)	1.05(4)
C18	H18c	1.067(12)	1.061(16)	1.07(5)	1.05(5)	1.066(12)	1.058(14)	1.06(4)	1.00(4)
C20	H20a	1.094(12)	1.057(16)	1.14(3)	1.04(7)	1.095(11)	1.056(13)	1.15(3)	1.03(4)
C20	H20b	1.093(13)	1.072(17)	1.04(5)	1.04(5)	1.096(12)	1.085(15)	1.05(4)	1.10(5)
C20	H20c	1.057(13)	1.062(15)	1.07(5)	1.29(9)	1.056(12)	1.064(12)	1.04(4)	1.05(7)
Average		1.080(12)	1.065(16)	1.08(4)	1.09(6)	1.080(12)	1.066(14)	1.08(4)	1.03(5)

S8 Intermolecular interactions energies of 2°AP-β

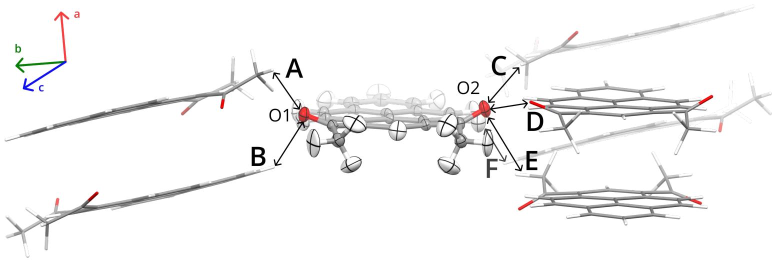

Figure S8.1: *Scheme of intermolecular interactions in the structure of 2°AP-β with symbols used in Table below.*

Table S8.1: *Total energies of intermolecular interactions in the structure of 2°AP-β, calculated in CrystalExplorer17[Mackenzie et al., 2017].*

Interaction	Symmetry operation	Interaction energy [kJ/mol]					
		100 K	150 K	190 K	250 K	293 K	350 K
Dimer	-x, -y, -z	-75.5	-74.8	-74.1	-73.9	-73.6	-71.1
Between dimers	-x, -y, -z	-63.2	-63.2	-62.9	-62.7	-63.4	-63.6
A	-x, y+1/2, -z+1/2	-22.5	-22.3	-22	-21.6	-20.5	-19.5
B	-x, y+1/2, -z+1/2	-7.1	-6.9	-6.9	-6.8	-6.9	-6.4
C	x, -y+1/2, z+1/2	-11.8	-11.8	-11.7	-11.2	-10.8	-10.2
D	x, y, z	-12.0	-11.6	-11.3	-10.7	-9.9	-8
E	-x, -y, -z	-12.8	-12.2	-11.7	-11.7	-10.8	-9.8
F	-x, y+1/2, -z+1/2	-7.5	-7.5	-7.5	-7.4	-7.6	-8.3

S9 The lowest-frequency lattice vibration modes for 2°AP-β

Table S9.1: *The lowest-frequency vibrational modes calculated for 2°AP-β with CRYSTAL17 [Dovesi et al., 2018]. Modes most related to the transverse vibrations of the carbonyl groups represented in bold.*

mode	Eigenvalue (Ha^2)	Freguencies (cm^{-1})	(THz)	Symmetry	IR Intensity	(km/mol)	Raman Response	(Arb)
1	0.6223E-17	0.0000	0.0000	(Bu)	A	0.00	I	0
2	0.7034E-16	0.0000	0.0000	(Bu)	A	0.00	I	0
3	0.2436E-15	0.0000	0.0000	(Au)	A	0.00	I	0
4	0.9627E-08	21.5340	0.6456	(Bg)	I	0.00	A	0.18
5	0.1839E-07	29.7631	0.8923	(Bg)	I	0.00	A	0.38
6	0.1945E-07	30.6072	0.9176	(Ag)	I	0.00	A	2.64
7	0.2046E-07	31.3952	0.9412	(Bu)	A	0.81	I	0
8	0.2197E-07	32.5310	0.9753	(Au)	A	0.23	I	0
9	0.3490E-07	41.0017	1.2292	(Bg)	I	0.00	A	0.16
10	0.3553E-07	41.3670	1.2402	(Au)	A	0.00	I	0.00
11	0.3689E-07	42.1560	1.2638	(Ag)	I	0.00	A	0.37
12	0.4573E-07	46.9356	1.4071	(Bg)	I	0.00	A	0.37
13	0.4776E-07	47.9645	1.4379	(Ag)	I	0.00	A	0.09
14	0.4842E-07	48.2968	1.4479	(Au)	A	2.32	I	0
15	0.4950E-07	48.8320	1.4639	(Bu)	A	3.39	I	0
16	0.6691E-07	56.7704	1.7019	(Ag)	I	0.00	A	2.28
17	0.9192E-07	66.5398	1.9948	(Bg)	I	0.00	A	11.92
18	0.9493E-07	67.6218	2.0273	(Bu)	A	11.08	I	0
19	0.1018E-06	70.0397	2.0997	(Au)	A	4.45	I	0
20	0.1121E-06	73.4887	2.2031	(Ag)	I	0.00	A	10.23
21	0.1232E-06	77.0291	2.3093	(Ag)	I	0.00	A	4.29
**22**	0.1368E-06	**81.1709**	2.4334	**(Bg)**	I	0.00	**A**	**1.75**
23	0.1517E-06	85.4743	2.5625	(Au)	A	7.82	I	0
24	0.1657E-06	89.3484	2.6786	(Bu)	A	0.05	I	0
25	0.1881E-06	95.1975	2.8539	(Au)	A	0.01	I	0
26	0.2251E-06	104.1292	3.1217	(Au)	A	0.05	I	0
27	0.2254E-06	104.2029	3.1239	(Ag)	I	0.00	A	3.25
28	0.2316E-06	105.6323	3.1668	(Bg)	I	0.00	A	0.05
29	0.2319E-06	105.6900	3.1685	(Bu)	A	10.81	I	0
30	0.2460E-06	108.8452	3.2631	(Ag)	I	0.00	A	1.79
**31**	0.2569E-06	**111.2467**	3.3351	**(Bg)**	I	0.00	**A**	**0.01**
32	0.2767E-06	115.4450	3.4610	(Ag)	I	0.00	A	5.90
**33**	0.2799E-06	**116.1213**	3.4812	**(Bu)**	**A**	**51.57**	I	0
34	0.2958E-06	119.3680	3.5786	(Bu)	A	26.56	I	0
35	0.3687E-06	133.2577	3.9950	(Bg)	I	0.00	A	1.41
36	0.3691E-06	133.3361	3.9973	(Au)	A	7.66	I	0
**37**	0.3821E-06	**135.6625**	4.0671	**(Au)**	**A**	**3.70**	I	0
38	0.3968E-06	138.2478	4.1446	(Bu)	A	64.56	I	0
39	0.4230E-06	142.7413	4.2793	(Bg)	I	0.00	A	1.20
40	0.4490E-06	147.0678	4.4090	(Ag)	I	0.00	A	5.42
41	0.4571E-06	148.3822	4.4484	(Au)	A	2.26	I	0
42	0.4922E-06	153.9843	4.6163	(Ag)	I	0.00	A	0.43
43	0.5012E-06	155.3787	4.6581	(Bu)	A	8.58	I	0
44	0.5062E-06	156.1567	4.6815	(Bg)	I	0.00	A	4.51
45	0.6350E-06	174.8972	5.2433	(Bu)	A	9.54	I	0
46	0.6689E-06	179.5001	5.3813	(Ag)	I	0.00	A	1.58
47	0.6816E-06	181.1996	5.4322	(Au)	A	25.91	I	0
48	0.6934E-06	182.7516	5.4788	(Bg)	I	0.00	A	4.46
49	0.8059E-06	197.0269	5.9067	(Ag)	I	0.00	A	1.05
50	0.8221E-06	198.9952	5.9657	(Bu)	A	1.77	I	0
51	0.8290E-06	199.8288	5.9907	(Au)	A	18.84	I	0
52	0.8409E-06	201.2557	6.0335	(Bg)	I	0.00	A	0.22

S10 Differential Scanning Calorimetry

The DSC measurements for 2°AP-β were performed using Mettler-Toledo DSC1 STAR^e system at a heating rate of 10°C/min under a dry N_2 atmosphere and at a constant flow (60 ml/min) over a temperature range from 25 to -150°C and back (cooling) and then from 25 to 500°C. Obtained data were analyzed using the STARe software provided by Mettler Toledo. The total of 4.47 mg of freshly prepared crystals of 2°AP-β was accurately weighted into open standard 40 μl aluminum crucibles using Mettler-Toledo XS105 DualRange balance. The sample was tested against traces of 2°AP-α polymorph (green fluorescence) under the hand-held UV lamp. The DSC curves on cooling and heating were depicted in Figure S10.1. The major endothermic peak at 177.38°C represents the melting point of 2°AP-β and is 3°C higher then observed during melting at the hot-stage [Zwolenik et al., 2024] but within expected tolerance. The uneven shape of the DSC curves in this regime is attributable to sample being in the form of large blocks of crystals rather than fine powder.

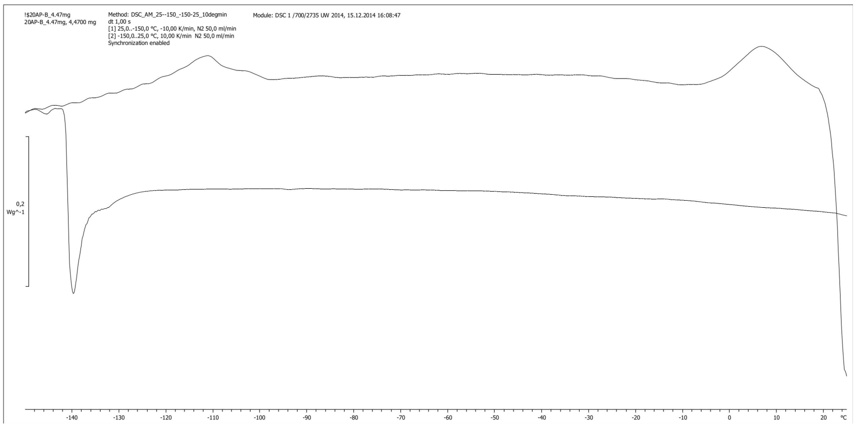

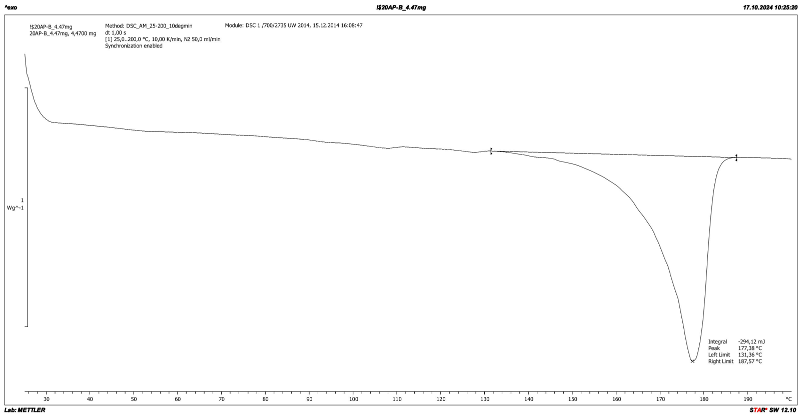

Figure S10.1: *The DSC curves of 2°AP-β on cooling (top) and heating (bottom).*

Chapter S2

Bibliography

[Allen and Bruno, 2010] Allen, F. H. and Bruno, I. J. (2010). Bond lengths in organic and metal-organic compounds revisited: X—H bond lengths from neutron diffraction data. *Acta Crystallographica Section B*, 66(3):380–386.

[Dovesi et al., 2018] Dovesi, R., Erba, A., Orlando, R., Zicovich-Wilson, C. M., Civalleri, B., Maschio, L., Rérat, M., Casassa, S., Baima, J., Salustro, S., and Kirtman, B. (2018). Quantum-mechanical condensed matter simulations with CRYSTAL. *WIREs Computational Molecular Science*, 8(4):e1360.

[Groom et al., 2016] Groom, C. R., Bruno, I. J., Lightfoot, M. P., and Ward, S. C. (2016). The Cambridge Structural Database. *Acta Crystallographica Section B*, 72(2):171–179.

[Kuhs, 1988] Kuhs, W. F. (1988). The Anharmonic Temperature Factor in Crystallographic Structure Analysis. *Australian Journal of Physics*, 41(3):369–382.

[Mackenzie et al., 2017] Mackenzie, C. F., Spackman, P. R., Jayatilaka, D., and Spackman, M. A. (2017). CrystalExplorer model energies and energy frameworks: extension to metal coordination compounds, organic salts, solvates and open-shell systems. *IUCrJ*, 4(5):575–587.

[Meindl and Henn, 2008] Meindl, K. and Henn, J. (2008). Foundations of residual-density analysis. *Acta Crystallographica Section A*, 64(3):404–418.

[Zwolenik et al., 2024] Zwolenik, A., Tchoń, D., and Makal, A. (2024). Evolution of structure and spectroscopic properties of a new 1,3-diacetylpyrene polymorph with temperature and pressure. *IUCrJ*, 11(4):519–527.
